# Nesprin-2 contains BH3-like motifs that can promote cell death

**DOI:** 10.1038/s41420-025-02534-5

**Published:** 2025-06-03

**Authors:** Hila Zohar, Amit Kessel, Liora Lindenboim, Dang Nguyen, Nir Ben-Tal, Gregg G. Gundersen, Howard J. Worman, David W. Andrews, Reuven Stein

**Affiliations:** 1 Department of Neurobiology, School of Neurobiology, Biochemistry and Biophysics, George S. Wise Faculty of Life Sciences, Tel Aviv University, Tel Aviv, 69978 Israel; 2 Department of Biochemistry and Molecular Biology, School of Neurobiology, Biochemistry and Biophysics, George S. Wise Faculty of Life Sciences, Tel Aviv, Israel; 3 Department of Medical Biophysics, Faculty of Medicine, University of Toronto, Toronto, ON Canada; 4 Biological Sciences Platform, Sunnybrook Research Institute, Toronto, ON Canada; 5 Department of Pathology and Cell Biology, Vagelos College of Physicians and Surgeons, Columbia University, New York, NY 10032 USA; 6 Department of Medicine, Vagelos College of Physicians and Surgeons, Columbia University, New York, NY 10032 USA; 7 Department of Biochemistry, Faculty of Medicine, University of Toronto, Toronto, ON Canada

**Keywords:** Apoptosis, Actin

## Abstract

BH3-only proteins are a subgroup of the pro-apoptotic Bcl-2 family proteins. They initiate apoptosis by interacting with the multidomain pro- and anti-apoptotic Bcl-2 family proteins. *SYNE2* encodes multiple nesprin-2 (Nes2) isoforms of which the most abundant and the largest is the nuclear envelope protein nesprin-2 giant (Nes2G). Nes2G is a component of the nuclear envelope Linker of Nucleoskeleton and Cytoskeleton (LINC) complex that connects the nucleus and the cytoskeleton. Previously, we showed that Nes2 has pro-apoptotic activity. We now show that Nes2G can bind multidomain pro-apoptotic and anti-apoptotic Bcl-2 family proteins and contains two BH3-like motifs near its N- and C-termini. Molecular modeling predicts that these BH3-like motifs have amphipathic α-helix structures and can dock onto the canonical groove of Bax and anti-apoptotic proteins as well as the trigger site of Bax. A chimeric tBid with its BH3 domain replaced with the C-terminal Nes2 BH3-like domain binds to Bax in cells. Furthermore, Nes2 BH3-like motif-containing fragments from the N- and the C-termini bind both pro-apoptotic and anti-apoptotic Bcl-2 proteins and promote cytochrome *c* release (indicative of apoptosis). Our results suggest that Nes2 acts as a BH3-only protein that regulates apoptosis by binding to the multidomain Bcl-2 family proteins.

## Introduction

Apoptosis is a regulated cell death process. A major apoptotic pathway is the intrinsic route, which leads to mitochondrial outer membrane (MOM) permeabilization (MOMP) and the release of mitochondrial apoptogenic factors such as cytochrome *c*. This, in turn, propagates cell death via caspase activation. The intrinsic pathway is regulated by the Bcl-2 protein family, which is comprised of anti-apoptotic proteins (e.g., Bcl-2, Bcl-x_L_ and Mcl-1) that inhibit apoptosis by interacting and sequestering their pro-apoptotic counterparts, the effector proteins Bax and Bak (also known as “executioners”), and the upstream initiators of the pathway, the BH3-only proteins (e.g., Bim, Bid, and Bad) (for review see [1–6] and references therein). MOMP is caused by the generation of pores in the MOM by the effector proteins following their activation by the BH3-only proteins. The latter can induce Bax/Bak activation indirectly by binding and neutralizing the anti-apoptotic Bcl-2 proteins and some (e.g., Bid and Bim) can also bind to and directly activate Bax and Bak.

The BH3-only proteins act via their BH3 domain, an amphipathic helix that contains four hydrophobic residues located at conserved positions (termed h1–h4) that bind to the hydrophobic groove of the anti-apoptotic and pro-apoptotic effector proteins. The helix also contains a conserved aspartate between h3 and h4 (h3 + 2), forming a salt bridge with a conserved arginine residue in the hydrophobic groove of effector proteins [7–9]. Two BH3 domain binding sites have been proposed to drive Bax activation [10, 11], the canonical groove [12, 13], which resembles BH3-domain binding groove of Bak [14, 15] and the anti-apoptotic Bcl-2 family proteins [16], as well as a trigger site or rear pocket, involving α1/α6 helices [17, 18]. Binding of the BH3 domains of the activating BH3-only proteins to the Bax canonical groove promotes unfolding of Bax (or Bak) into “core” (α2–α5) and “latch” (α6–α8) domains. This leads to the generation of the basic unit “symmetric dimers” [12, 13] and the subsequent generation of homo-oligomers and membrane pores [19, 20]. These conformation changes also involve exposure of the N-terminus of the effector proteins. In addition, it has been suggested that binding of activating BH3 domains to the trigger site of Bax leads to displacement of the α9 helix from the hydrophobic groove and translocation to the mitochondrial membrane through allosteric changes [17, 21].

Nesprin-2 (Nes2) is a member of the superfamily of spectrin repeat (SR) containing proteins. It was initially identified as the outer nuclear membrane component of the Linker of Nucleoskeleton and Cytoskeleton (LINC) complex [22]. Nes2 contains a Klarsicht/ANC-1/Syne-1 homology (KASH) domain at its C-terminus that projects into the perinuclear space, where it interacts with the Sad1/UNC84 (SUN) domain of SUN proteins, integral proteins of the inner nuclear membrane and the other component of the LINC complex. The N-terminus of Nes2 extends into the cytoplasm where it interacts with cytoskeletal elements such as actin microfilaments and microtubules, thus linking the cytoskeleton to nuclear lamina (for reviews see [23–31]).

The Nes2 encoding gene, *SYNE2*, contains more than 100 exons encoding multiple isoforms that arise by alternative RNA splicing and multiple transcriptional initiation sites [32–34]. The largest isoform of Nes2 is termed nesprin-2-giant (Nes2G). The N- and C- terminal domains of Nes2G are connected by a rod-like structure composed of multiple SRs. The structure of a single SR is characterized by three bundled antiparallel α-helices separated by two loop regions. Consecutive SRs are linked by an α helix region which connects the last helix of one repeat with the first helix of the adjacent one [35]. SRs mediate protein-protein interactions, for example Nes2G’s SR11-12 bind to FHOD1 [36] and its SR51-53 bind to telethonin and four-and-half LIM domain-2 [37].

Recently, we found that Nes2 can act as an apoptotic mediator via Bcl-2 family proteins and involves direct binding to Bax, activation of Bak, as well as inhibition of Bcl-x_L_ anti-apoptotic activity [38, 39]. These findings suggest that Nes2 regulates apoptosis by affecting both anti-apoptotic and pro-apoptotic Bcl-2 proteins. To further explore the pro-apoptotic mechanism of action of Nes2 we focused on its interactions with Bcl-2 proteins. We show that Nes2 can bind to both anti- and pro- apoptotic proteins and that this binding can be mediated by its two BH3-like motifs. Furthermore, Nes2 BH3-like motif-containing fragments can promote apoptosis. Collectively, our results suggest that Nes2 regulates apoptosis by acting as a BH3 only protein.

## Results

### Nes2G interacts with both anti- and pro- apoptotic Bcl-2 proteins

**Fig. 1:**
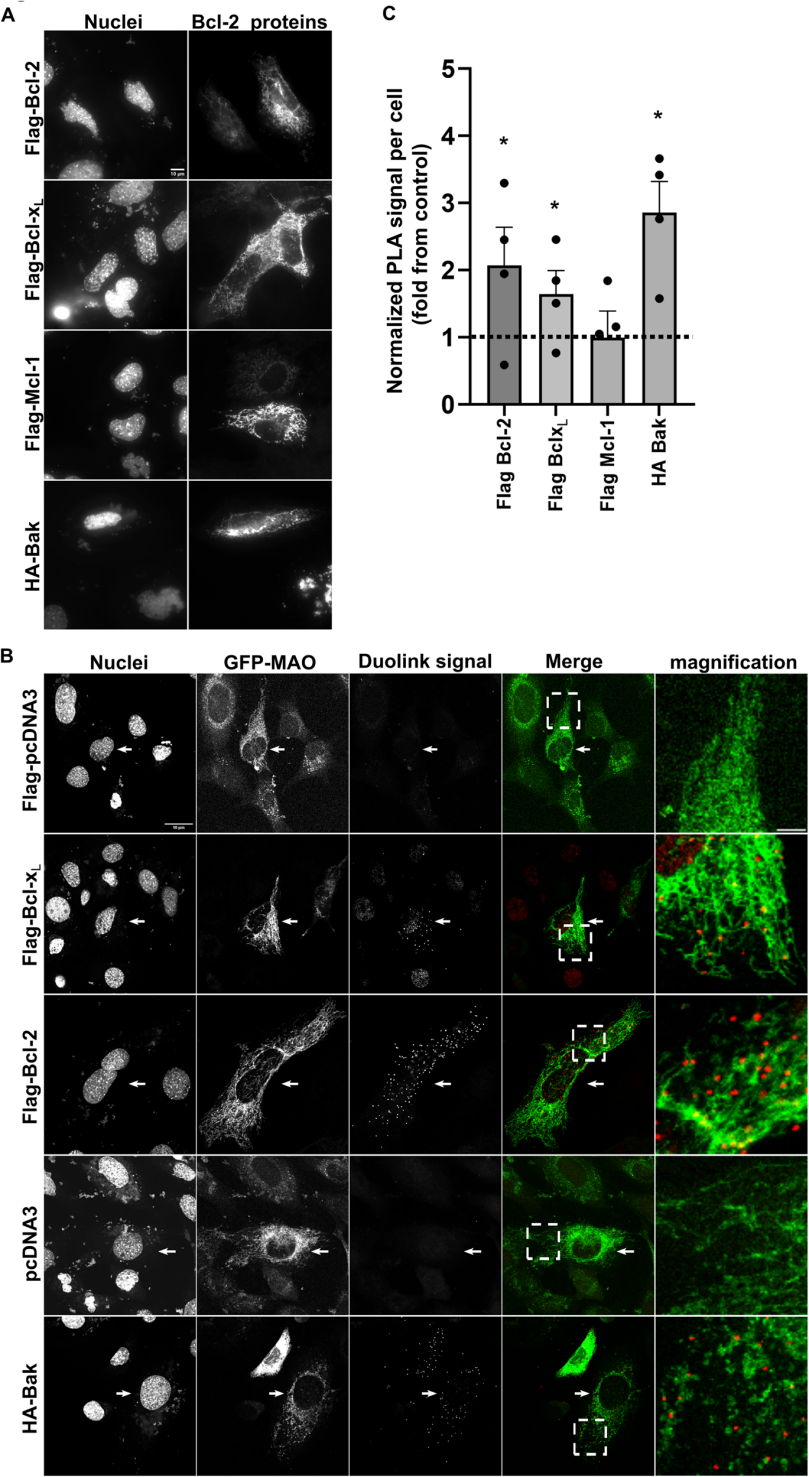
Nes2G interacts with anti- and pro-apoptotic members of the Bcl-2 family proteins. **A** Representative IF photomicrographs of *Casp9*^−/−^ MEFs expressing the different transfected Bcl-2 family proteins from 2 independent experiments. Nuclei are stained with Hoechst 33258 dye. Bar = 10 µm. **B** Representative confocal fluorescence photomicrographs of GFP-MAO-expressing *Casp9*^−/−^ MEFs showing Duolink-PLA signal. Bar = 10 µm. The photomicrographs show the same field visualized separately for nuclei (DAPI), transfected cells (GFP-MAO) and Duolink signal (appears as dots). Bar = 10 µm. Merge images of the Duolink-PLA signal (red) and GFP-MAO (mitochondria; green) are shown in the two right panels. Higher magnification of a representative area (denoted by a box) in the merge images illustrates the presence of Duolink dots in close proximity to mitochondria. The dashed white box indicates the area that was magnified. Bar = 2 µm. Arrows indicate representative cells. **C** Quantification of the Duolink-PLA signal in GFP-MAO transfected cells. The cells were imaged and Duolink dots in individual transfected cells were counted. The values obtained for each cell were normalized to its size (number of dots per cell area). The results are expressed as the ratio between the average of the normalized Duolink-PLA signal in cells transfected with the indicated Bcl-2 family proteins relative to the average of normalized Duolink-PLA signal of their corresponding control (pcDNA3 for Bak and FLAG-pcDNA3 for the anti-apoptotic proteins). The results are presented as mean ± SEM (*n* = 4) with each black circle indicating an individual experiment. *p < 0.05, paired two-sample Student’s *t* test rela*t*ive to corresponding control was performed after Log transformation for each treatment (Dashed line: ratio = 1).

We used Duolink-PLA to assess the ability of endogenous Nes2G to interact with exogenously expressed Bcl-x_L_, Bcl-2, Mcl-1 (anti-apoptotic) and Bak (pro-apoptotic) Bcl-2 family proteins. We transiently transfected *Casp9*^*−/−*^ MEFs (to attenuate cell death) with expression vectors to express Flag-Bcl-x_L_, Flag-Bcl-2, Flag-Mcl-1 and HA-Bak, and validated their expression by immunofluorescence microscopy using anti-FLAG or anti-HA Abs, respectively. As expected for anti-apoptotic proteins and Bak that mainly reside in the mitochondria, the transfected proteins exhibited a web-like expression pattern (Fig. 1A). Next, we transfected the *Casp9*^*−/−*^ MEFs with each of the above-mentioned vectors as well as with pcDNA3 or Flag-pcDNA3 (control empty vectors), together with a GFP-monoamine oxidases (MAO) (which marks the mitochondrial outer membrane) expression vector, to identify transfected cells and evaluate the location of the interaction relative to mitochondria. Twenty-four hours after transfection, we performed Duolink-PLA using anti-nes2G Abs [40] together with anti-HA or anti-Flag Abs. Cells transfected with the control vectors contained only a low amount of Duolink-PLA signal (dots); however, cells transfected with Bcl-x_L_, Bcl-2 and Bak, but not with Mcl-1, contained a significantly higher Duolink signal (1.8–2.8-fold increase, Fig. 1B, C). Inspection of the location of the Duolink dots relative to the mitochondria (GFP-MAO signal) showed that some of the dots localized near the mitochondria. These results suggest that Nes2G can interact with both anti- (Bcl-2 and Bcl-x_L_) and pro- (Bak) apoptotic Bcl-2 family proteins and that some of the interactions occur in close proximity to mitochondria.

### Identification of BH3-like motifs in Nes2

Our current and previous findings showing that Nes2G can interact with both anti-apoptotic (Bcl-2 and Bcl-x_L_) and executioner pro-apoptotic (Bak and Bax [39]) Bcl-2 family proteins suggest that Nes2G may function similarly to BH3-only proteins known to act via their BH3 motifs [1, 2]. Therefore, we next examined if Nes2 contains BH3-like motif(s).

**Fig. 2:**
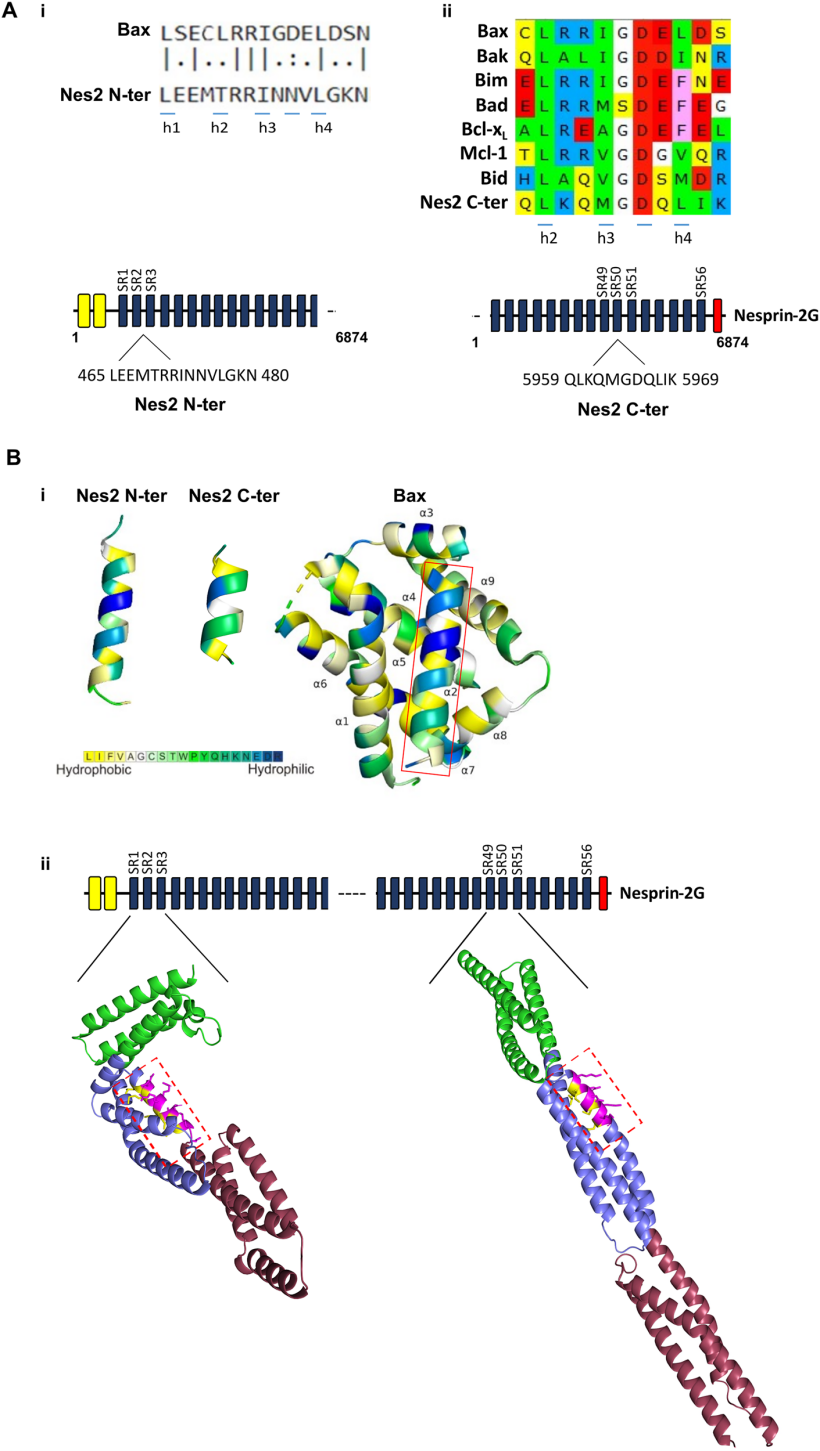
Nes2G contains BH3-like motifs within the N-terminal and C-terminal regions. **A** (i) The upper panel shows pairwise sequence alignment of the Bax-BH3 domain and the Nes2G BH3-like motif within Nes2 N-terminal (Nes2 N-ter) region. Identical residues among the two sequences are indicated by vertical lines, whereas similar residues are indicated by dots (two dotes represent higher similarity than one dot). The lower panel shows schematic representation of Nes2G domains and the location and amino acid sequence of the Nes2 N-ter motif. The calponin homology domains at the N-terminus are represented by yellow boxes, the spectrin repeats (SR) are represented by dark blue boxes. (ii) The upper panel shows multiple-sequence alignment of BH3 sequences from the indicated Bcl-2 proteins with the Nes2G BH3-like motif within Nes2G C-terminal (Nes2 C-ter) region. Positively charged residues are colored red, negatively charged residues are colored blue, nonpolar residues are colored green, polar uncharged residues are colored yellow and aromatic residues are colored pink. h1-4 (in i and ii) indicate the positions of the four consensuses nonpolar residues of BH3 domains. The lower panel shows schematic representation of Nes2G domains and the location and amino acid sequence of the Nes2 C-ter motif. The SR are represented by dark blue boxes and the C-terminal KASH domain is represented by the red box. The UniProt accession numbers used for pairwise or multiple sequence alignments are: Bax (Q07813), Bak (O08734), Bim (O54918), Bad (Q61337) Bid (P70444), Bcl-x_L_ (Q64373), Mcl-1 (P97287), nesprin-2 (Q6ZWQ0). **B** (i) Predicted structure and amphipathicity of Nes2 N-terminal and C-terminal motifs. Note the similarity in structure and amphipathicity between Bax-BH3 within the structure of Bax (PDB ID: 5W62) (boxed) and the predicted Nes2 BH3-like motifs. The structures are colored by the Kessel-Ben-Tal hydropathy scale [47], which ranges from polar (blue) to nonpolar (yellow; see color scale at the bottom). (ii) Predicted structure and amphipathicity of Nes2 N-ter and C-ter motifs within their indicated spectrin repeats (SR 1-3 or SR 49-51, for Nes2 N-ter and Nes2 C-ter motifs, respectively). The Nes2 BH3-like motifs are marked by dashed red boxes; the N-ter motif is localized at the end of SR2 and the beginning of SR3 and the C-ter motif in SR50. The different SRs are colored in green (SR1, SR49), purple (SR2, SR50) and Boudreaux (SR3, SR51). Note the amphipathic α-helical structure of both BH3-like motifs (nonpolar residues are yellow; polar residues are pink).

To this end, we performed a pairwise alignment of the amino acid sequences of BH3 domains of representative pro-apoptotic Bcl-2 proteins: Bax, Bak, Bim or Bad, each against the full-length sequence of mouse nesprin-2 using EMBOSS Needle [41, 42]. This analysis identified a stretch of 15 amino acids in Nes2G (position 465-480), located at the end of SR2 and the beginning of SR3, showing 50% sequence identity with the Bax-BH3 domain, which we termed Nes2-N-terminal motif (Fig. 2Ai). Notably, the amino acids of the Nes2 N-terminal motif at the critical positions h1, h3 and h4 were identical to that of Bax’s BH3 at these positions (L, I and L, respectively). At position h2, Nes2 N-terminal motif contains threonine instead of leucine. Both amino acids have branched side chains, and while threonine, unlike leucine, is polar, its side chain is still able to form nonpolar interactions. At position h3 + 2, Nes2 N-terminal motif contains asparagine instead of aspartic acid (Fig. 2Ai). We also performed a multiple sequence alignment of the Nes2 amino acid sequence with the BH3 domain sequences of Bax, Bak, Bim, Bad, Bid, Bcl-x_L_, and Mcl-1, using the MAFFT software [43, 44] to determine if Nes2 contains a segment that is homologous to a shared profile of all of these BH3 domains. This analysis identified an additional BH3-like motif in Nes2, eleven amino acids long, located at the C-terminus, in the middle of SR50, at position 5959-5969 (Fig. 2Aii). In this motif, the h2 position contains the conserved leucine residue and the h3 and h4 positions contain nonpolar amino acids like other BH3 domains. The motif also contains the conserved aspartic acid at the h3 + 2 position. We termed this motif Nes2-C-terminal.

Next, we asked if the structure of the putative Nes2 BH3-like motifs resemble the canonical BH3 motifs found in Bcl-2 family proteins. Since the structure of these regions of Nes2 have not been experimentally determined, we used AlphaFold2 [45], through the ColabFold web server [46], to predict the structures of its two putative BH3 motifs as well as the structures of SR1-3 and SR49-51 that contain the BH3-like motifs. The predicted structures of both putative Nes2 BH3-like motifs were α-helical and amphipathic [47], closely resembling Bax-BH3 (Fig 2Bi). When the structures of these helices were superposed onto the structure of the BH3 helix of Bax, their polar residues projected towards the surrounding solvent, whereas their nonpolar residues faced the nonpolar pocket of the protein. The predicted structure of both SR1-3 and SR49-51 adopted the characteristic SR architecture (Fig. 2Bii) in which the Nes2 BH3-like motifs within them retained their α-helical structure and amphipathicity; the hydrophobic side chains face inward and hydrophilic side chains facing outward (Fig. 2Bii, dashed boxes).

**Fig. 3:**
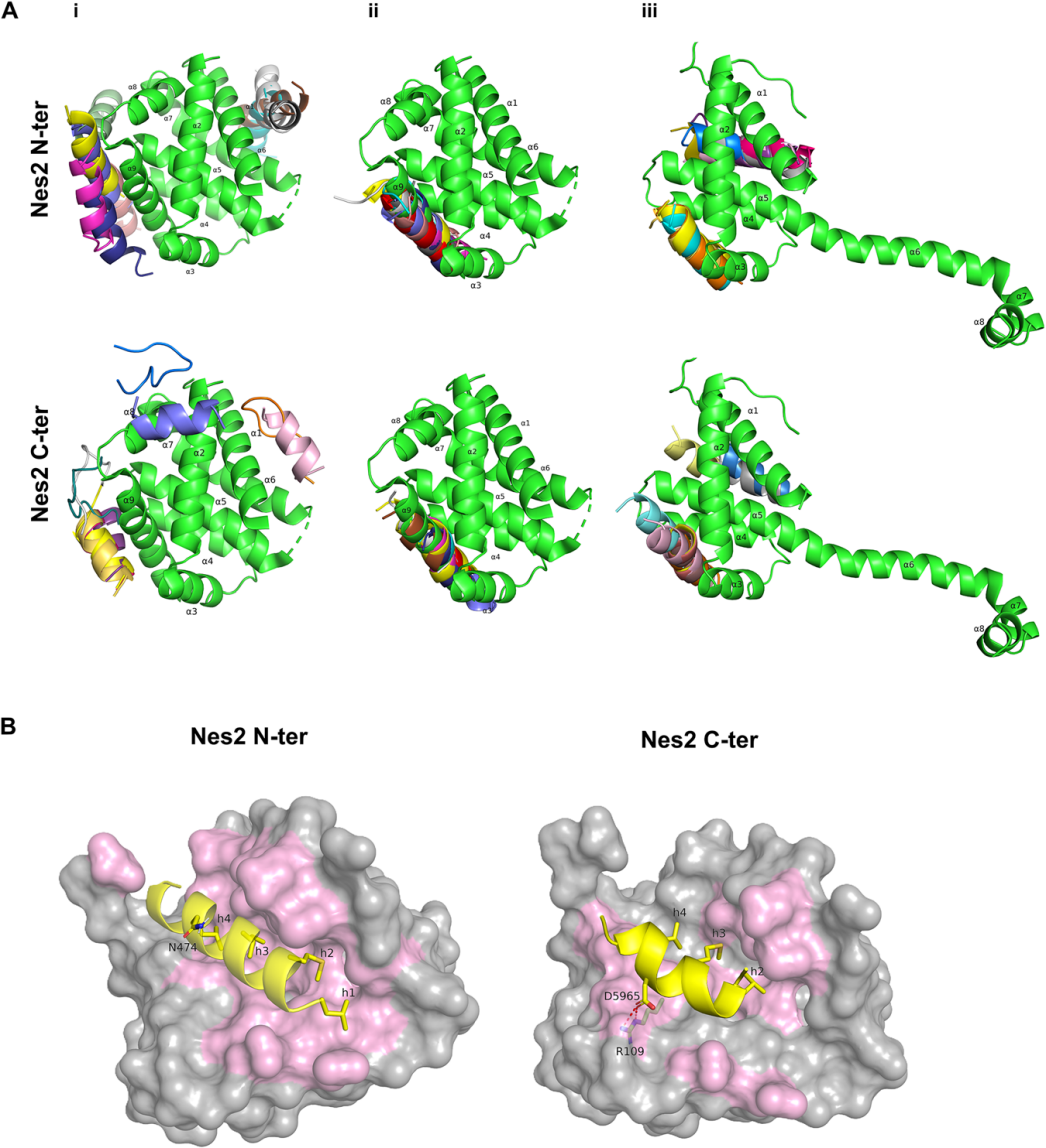
Predicted interactions of Nes2 BH3-like motifs with Bax. **A** Models of Nes2 BH3-like motifs docking onto three different structures of Bax, as follows: (i) Full-length Bax (PDB: 5W62); (ii) ΔC Bax (PDB: 5W62, after removal of α9 from the structure for the docking). Note that the Bax structure shown contains α9 only to illustrate the position of the Nes2 BH3 motif in Bax relative to the position of α9; (iii) One monomer of Bax swapped dimer (PDB: 4BD2) (BaxΔC21 in complex with BidBH3 after removal of the Bid-BH3 from the structure). In each complex, Bax is colored green with its α-helices numbered and the positions of the top ten docking solutions for Nes2 N-terminal (Nes2 N-ter) and Nes2 C-terminal (Nes2 C-ter) motifs are shown. Each docking solution is colored by a different color. **B** Residues in the Nes2 BH3-like motifs that interact with the Bax canonical hydrophobic pocket (PDB: 5W62, after removal of α9 from the structure). A representative docking solution is shown for each Nes2 motif (colored yellow). Depicted are h1-h4 or h2-h4 positions (for Nes2 N-ter and Nes2 C-ter, respectively), h3 + 2 position (occupied by N474 in Nes2 N-ter and D5965 in Nes2 C-ter motif), the arginine residue in the Bax hydrophobic groove (R109) and the salt bridge between Nes2 D5965 and Bax R109. The molecular surface structure of Bax is shown to emphasize its geometric compatibility with the Nes2 interacting residues. Amino acids of the hydrophobic groove that are predicted to interact with Nes2 motifs (lie within 4 Å of Nes2 residues) are colored pink (backbone and sidechain of the interacting amino acids).

We asked if the Nes2 BH3-like motifs might interact with Bcl-2 family proteins. To do so, we performed global (blind) molecular docking simulations of the Nes2 N-terminal and Nes2 C-terminal helices onto Bcl-2 family proteins using PatchMAN [48]. Given a peptide sequence and a three-dimensional structure of a target protein, PatchMAN predicts the most likely binding mode of the two, as well as the structure of the bound peptide. Docking simulations were performed with Nes2’s N-terminal and C-terminal sequences, using available experimental structures of Bax: globular Bax (PDB ID: 5W62) or Bax ΔC21 swapped dimer complexed with BidBH3 (PDB ID: 4BD2), from which we used one monomer of the symmetrical dimer, after removing the Bid-BH3 peptide from the structure. The simulations suggested that each of the putative Nes2 BH3-like motifs can dock near the canonical pocket (helices α2-α5) of globular Bax, despite the presence of α9 in the pocket (Fig. 3Ai). Notably, the two BH3-like motifs also docked within Bax’s trigger pocket (helices α1 and α6) [17]. When we removed α9 from Bax structure, the two Nes2 BH3-like motifs were predicted to dock only within the canonical pocket, at the exact space which was previously occupied by α9 (Fig. 3Aii; for illustration, α9 is included in the image).

Molecular docking simulations of Nes2 BH3-like motifs on BaxΔC21:BidBH3 complex (PDB ID: 4BD2), which exhibit opening of the latch domain (α6, α7 and α8) and domain-swap [12], suggested that both motifs dock within the canonical pocket, and also at a rear site close to α1, at the position inhabited by α6 in the structure of full-length globular Bax (PDB ID: 5W62) (Fig. 3Aiii). We also examined the noncovalent interactions between specific positions of the Nes2 BH3-like motifs and Bax (PDB: 5W62, after removal of α9) in the PathMAN-predicted binding poses. In the predicted binding poses, the nonpolar residues at positions h1-h4 in Nes2 N-terminal and h2-h4 in Nes2 C-terminal project into the hydrophobic groove of Bax, where they form nonpolar interactions with the pocket (Fig. 3B). In addition, the aspartate residue located at the h3 + 2 position of the Nes2 C-terminal motif is predicted to form a salt bridge with the arginine residue of Bax that lies within the hydrophobic pocket, similar to the salt bridge formed between the Bid-BH3 aspartate and the BaxΔC21 arginine residue [12]. However, the Nes2 N-terminal asparagine residue located at h3 + 2 position projects away from the pocket and thus is not predicted to interact with the arginine residue of Bax within the pocket.

**Fig. 4:**
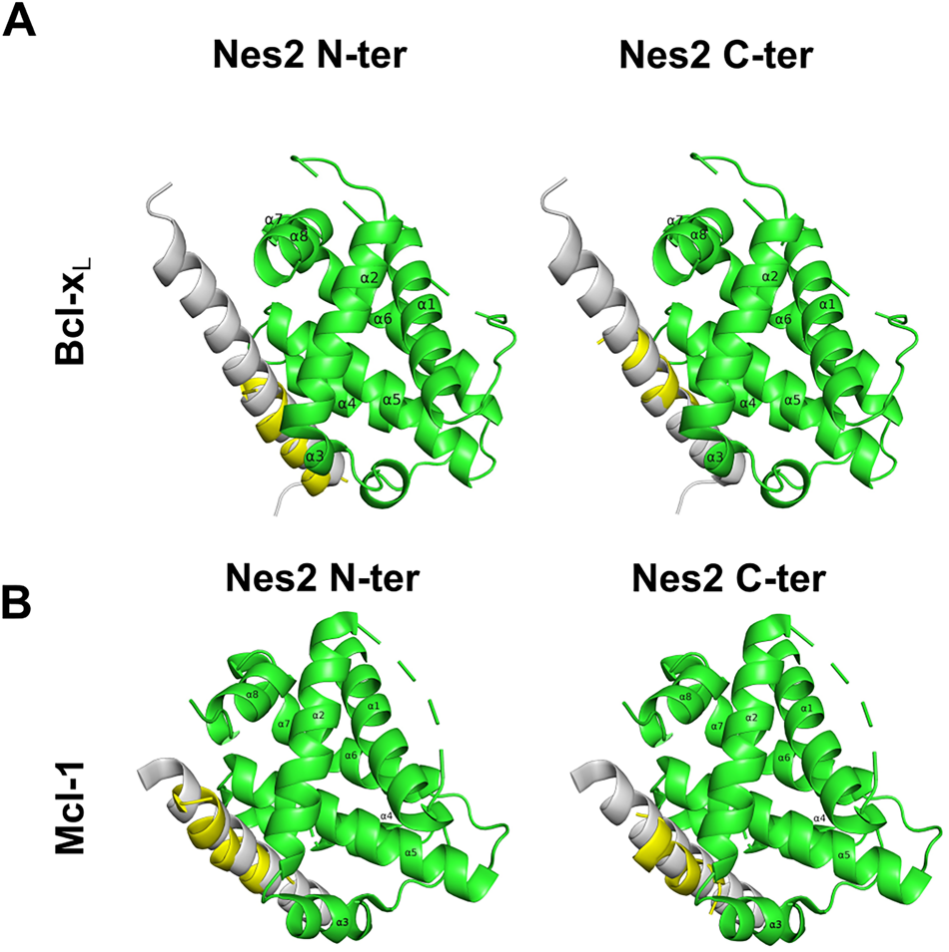
Predicted interaction of Nes2 BH3-like motifs with the hydrophobic groove of Bcl-x_L_ and Mcl-1. Docking-predicted complexes of the Nes2 BH3-like motifs with **A** the experimentally determined structure of Bcl-x_L_ complexed with BimBH3 (PDB: 1PQ1, BimBH3 was removed from the structure for docking) or with **B** the experimental structure of hMcl-1^BLR^ complexed with hBim BH3 (PDB: 2NL9, BimBH3 was removed from the structure for docking). Bcl-x_L_ and Mcl-1 are shown in green, with their α-helices numbered. Nes2 N-ter and C-ter motifs are colored yellow. To illustrate the position of the Nes2 BH3 motifs relative to Bim BH3, the images also include the Bim BH3 peptide, colored gray. The results show the best docking solution of Nes2 motif for each protein.

Finally, we used modeling methods to determine if the Nes2 BH3-like motifs could potentially dock within the hydrophobic pocket of anti-apoptotic proteins, the site where they interact with the BH3 domains of their respective pro-apoptotic proteins. The analysis was performed on the structure of Bcl-x_L_ and Mcl-1 in complex with the BH3 motif of the BH3-only protein Bim: Bcl-x_L_:Bim complex (PDB ID: 1PQ1) and hMcl-1^BLR^:hBim complex (PDB ID: 2NL9). We removed the BimBH3 motif from each complex and performed molecular docking simulations of the interactions of the Nes2 BH3-like motifs with Bcl-x_L_ and Mcl-1. This analysis predicted that both Nes2 BH3-like motifs dock within the canonical hydrophobic pocket of both Bcl-x_L_ and Mcl-1, at the similar position and orientation as that of Bim-BH3 bound to these structures (Fig. 4).

### The Nes2 C-terminal BH3-like motif can bind Bax in live cells

**Fig. 5:**
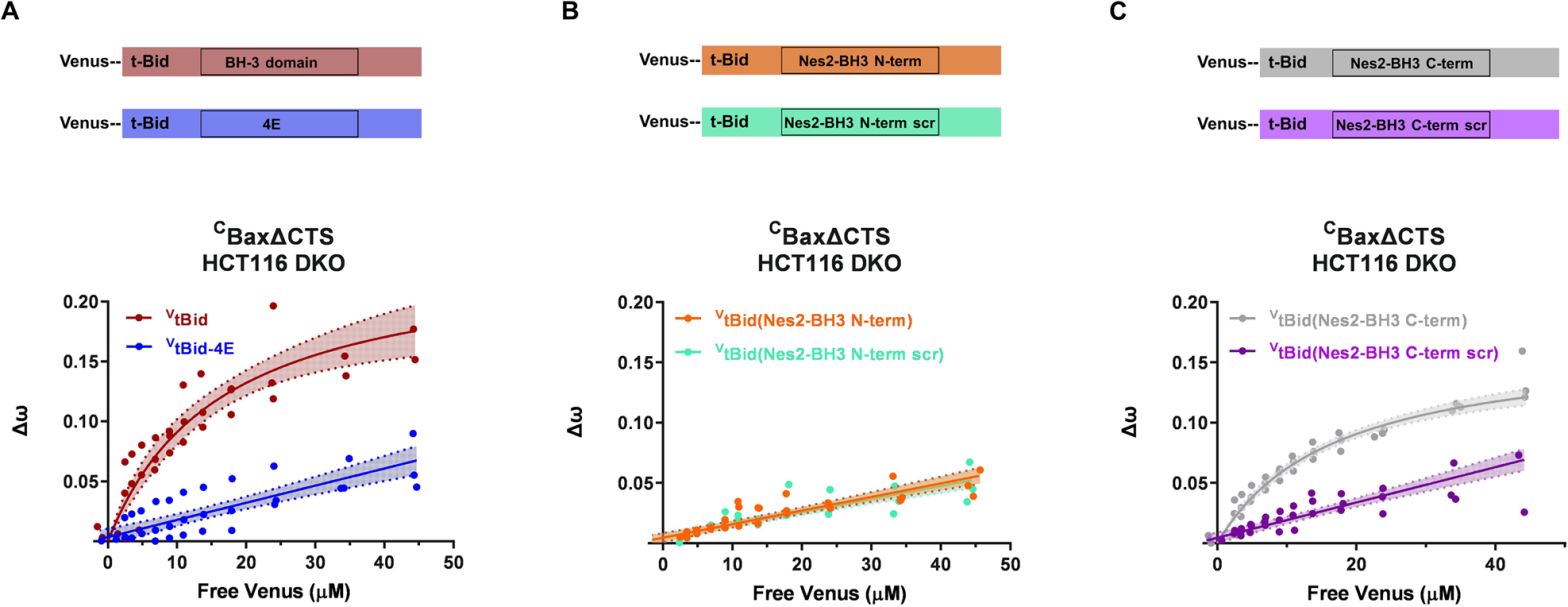
Nes2 C-terminal BH3 interacts with CTS-truncated Bax in live cells. Quantitative Fast FLIM-FRET (qF3) was used to measure binding of ^V^tBid to ^C^BaxΔCTS in HCT116 DKO cells. Δω on the *y*-axis represents the changes in angular frequency on a phasor plot and corresponds to the extent of fluorescence resonance energy transfer (FRET) between the donor protein mC3 and the acceptor protein Venus. **A** FRET data (Δω) for ^V^tBid binding to ^C^BaxΔCTS (red dots indicate individual experiments) was fit to the Hill equation (red binding curve with 95% confidence interval for the best fit shown by the shaded area and dotted lines). A curve approaching saturation as free Venus concentration increases above 30 µM indicates binding. Assuming saturation at above 40 μM free Venus using the nonlinear regression method to minimize the residual sum-of-squares (GraphPad Prism 9.5.0) (the slight upward trend presumably representing background collisions) resulted in an apparent Kd for ^V^tBid binding to ^C^BaxΔCTS of 16.3 µM. FRET data (Δω) for the negative control, ^V^tBid-BH3-4E, and ^C^BaxΔCTS (in blue), is best fit to a straight line as free Venus concentration increases, indicative of collisions between the donor and acceptor fluorophores rather than protein binding. **B** FRET data (Δω) for ^V^tBid (Nes2-BH3 N-ter) (orange) and the scrambled control version ^V^tBid (Nes2-BH3 N-ter scr) (green) fit best to straight lines similar to the collision control ^V^tBid-4E in (**A**). This indicates that only collisions were detected for ^V^tBid (Nes2 BH3 N-ter) and ^C^BaxΔCTS. **C** FRET data (Δω) for ^V^tBid(Nes2-BH3 C-ter) (gray) indicates binding to ^C^BaxΔCTS with an apparent Kd of 17 µM. The FRET data for the negative control in which the C-terminal Nes2-BH3 sequence is scrambled ^V^tBid(Nes2-BH3 C-ter scr) (purple) fits a straight line indicates that in live cells, only collisions were detected for these chimeras. Schematic representation of each Venus-tBid constructs is indicated.

We next assessed if the N-terminal and C-terminal motifs in Nes2G could bind Bax in live cells using the quantitative fast fluorescence lifetime imaging microscopy-Förster resonance energy transfer (FLIM-FRET) (qF3) method [49]. We examined if these motifs could replace the BH3 motif of Bid and induce binding of a chimeric tBid(Nes2-BH3) to Bax. We transiently transfected HCT116 cells lacking Bax and Bak (DKO cells) to express acceptor fusion proteins in which Venus (V) was fused to the N-terminus of tBid (^V^tBid) or to chimeric tBid(Nes2-BH3). Immunoblotting confirmed that these cells stably express the donor protein mCerulean3 (C) fused to the N-terminus of α9-truncated Bax (^C^BaxΔCTS) (Supplementary Fig. S1). As a negative control, we assessed the binding of chimeric tBid containing a scrambled sequence of the corresponding Nes2 BH3-like motif - [^V^Bid(Nes2-BH3 N-ter-scr) or ^V^tBid(Nes2-BH3 C-ter scr) instead of tBid BH3. Additional negative controls included the non-binding version tBid with 4 glutamic acids substituting for the 4 key hydrophobic residues in the Bid BH3 (^V^Bid-BH3-4E) to measure collisions as opposed to binding interactions. WT ^V^tBid bound to ^C^BaxΔCTS as indicated by a binding curve that plateaued and fit to the hill-slope equation (Fig. 5A). The negative control, ^V^tBid-BH3-4E, did not bind to Bax as indicated by a linear fit to the data indicating increasing collisions between the two fluorophores as the acceptor Venus concentration increases. In this assay, ^C^BaxΔCTS bound to ^V^tBid(Nes2-BH3 C-ter) but not to ^V^tBid(Nes2-BH3 N-ter) (Fig. 5B, C).The apparent binding affinity (K_d_) obtained by fitting the data to a Hill equation with *h* = 1 for ^V^tBid(Nes2-BH3 C-ter) binding to ^C^BaxΔCTS was 17 μM, a value similar to the 16.3 μM value obtained for WT ^V^tBid binding to ^C^BaxΔCTS. The negative controls ^V^tBid-BH3-4E and the ^V^tBid mutants containing scrambled versions of the putative Nes2 BH3 sequences, ^V^tBid(Nes2-BH3 N-ter scr) and ^V^tBid(Nes2-BH3 C-ter scr), all gave S-ratios below 2. This suggested that the binding data are best modeled with linear regression and indicated that the increase in Δω as the amount of free acceptor (Venus) increases is due to collisions and not protein-protein binding interactions.

### BH3-like motif-containing Nes2 fragments can bind pro- and anti- apoptotic Bcl-2 family proteins

**Fig. 6:**
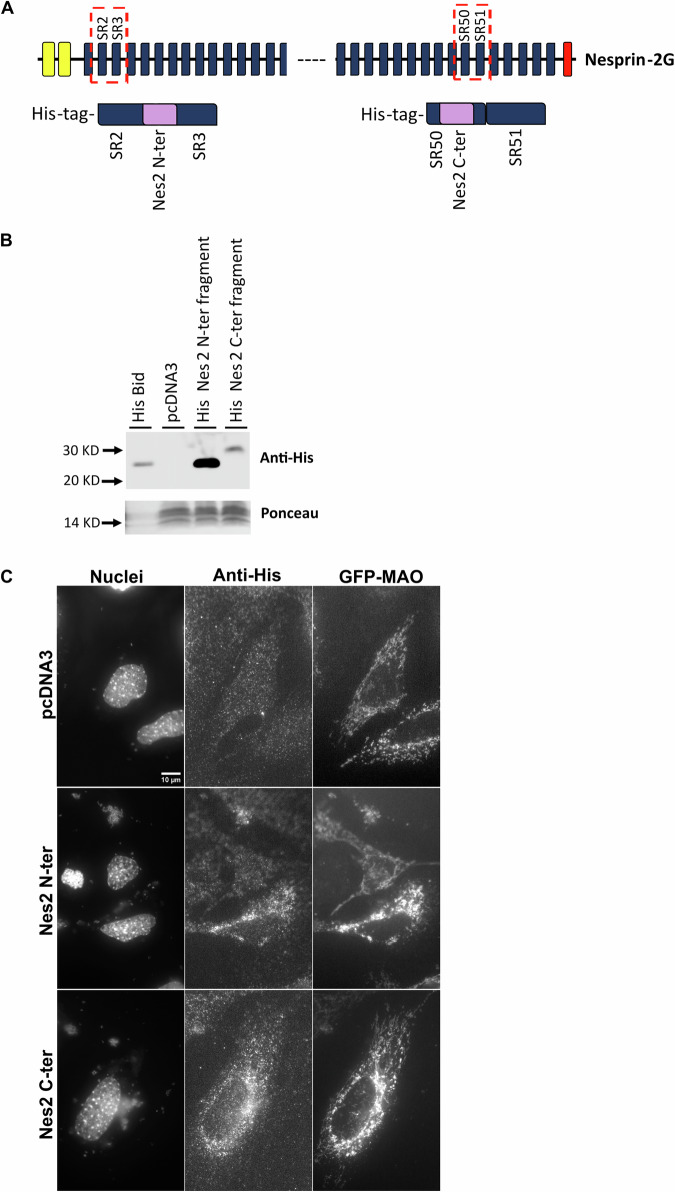
Expression of Nes2 N-terminal and Nes2 C-terminal fragments in cells. **A** Schematic representation of Nes2G depicting the position and content of the Nes2 N-terminal (Nes2 N-ter) and Nes2 C-terminal (Nes2 C-ter) fragments. The diagram shows the locations of the SRs in Nes2G (dashed boxes). The Nes2 N-ter and Nes2 C-ter fragments are comprised of SR2-3 and SR50-51, respectively, both tagged with His-tag at their N terminus. The position of Nes2 BH3-like motifs in the fragments is depicted in pink in the lower panel. **B** Expression of Nes2 N-ter and Nes2 C-ter fragments in 293T HEK cells. A representative immunoblot from two independent experiments of lysates prepared from 293T HEK cells transfected with Flag-pcDNA3 (negative control), or expression vectors encoding His-Nes2 N-ter or Nes2 C-ter fragments (with 20 µM Q-VD-OPH) as well as a lysate prepared from bacteria expressing His-Bid, probed with anti-His antibody (control for anti-His Ab reactivity). Ponceau staining was used as internal loading control. The expected molecular weights for His-Bid, Nes2 N-ter fragment and Nes2 C-ter fragment are ~22.5 KD, ~23 KD (205 amino acids) and ~27 KD (236 amino acids), respectively. The corresponding uncropped immunoblot is shown in Supplementary Fig. S4. **C** Expression of His-Nes2 N-ter and His-Nes2 C-ter fragments in *Casp9*^−/−^ MEFs. Representative IF micrographs (*n* = 2) of *Casp9*^−/−^ MEFs transfected with His-Nes2 N-ter or His-Nes2 C-ter expression vectors together with GFP-MAO stained with anti-His Ab and Hoechst dye (to detect nuclei). The IF micrographs show the same field visualized separately for Hoechst dye (Nuclei), His Ab (Nes2 fragment), and GFP-MAO labeling. Bar = 10 µm.

Having demonstrated that the Nes2 C-terminal motif can bind to Bax, at least in the context of tBid, we were interested in examining its potential cellular function in the context of its natural surrounding regions. Since the inability of ^V^tBid(Nes2-BH3 N-ter) to bind Bax may be context-dependent, we also examined the Nes2-BH3 N-terminal motif. To do so, we prepared expression vectors encoding His-tagged SR2-3 and SR49-50, the regions that contain and surround the Nes2 N-terminal and C-terminal BH3-like motifs, respectively (herein His Nes2 N-terminal and His Nes2 C-terminal fragments) (Fig. 6A). The expression of the corresponding Nes2 fragments was verified by immunoblotting of protein extracts prepared from 293 T HEK cells transfected with the expression vectors. Anti-His Abs detected bands of the predicted molecular mass (~23 kDa and ~27 kDa) corresponding to the His-Nes2 N-terminal (205 amino acids) and the His-Nes2 C-terminal (236 amino acids) fragments (Fig. 6B). The expression of these fragments was also verified in *Casp9*^*−/−*^ MEFs co-transfected with plasmids encoding the Nes2 fragment together with GFP-MAO (to identify transfected cells) by immunofluorescence (IF) staining against anti-His (Fig. 6C). These experiments confirmed the expression of these Nes2 fragments in transfected cells.

**Fig. 7:**
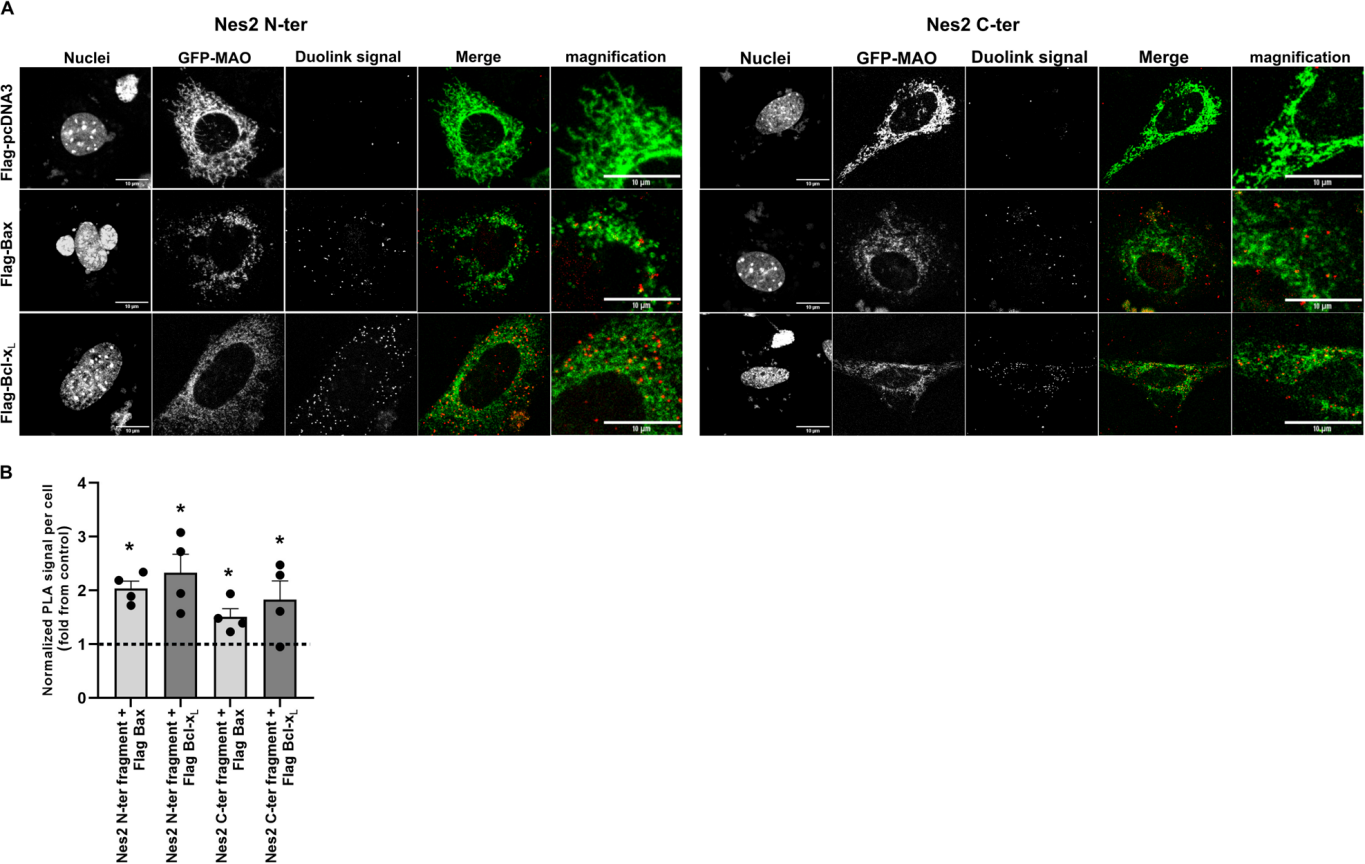
Nes2 N-terminal and Nes2 C-terminal fragments interact with Bax and Bcl-x_L_. **A** Representative confocal micrographs showing Duolink-PLA signals and mitochondrial distribution (indicated by GFP-MAO) in *Casp9*^−/−^ MEFs expressing either His-Nes2 N-terminal (Nes2 N-ter) or C-terminal (Nes2 C-ter) and either Flag-Bax, Flag-Bcl-x_L_ or Flga-pcDNA3 (control). Duolink-PLA was preformed using anti-His and anti-FLAG antibodies. The images represent the same field visualized separately for detecting nuclei (DAPI), transfected cells (GFP-MAO) and Duolink-PLA signal. Merge images of the Duolink-PLA signal (red) and GFP-MAO (mitochondria; green) are shown in the two right panels. Higher magnification of a representative area in the merge images illustrates close proximity of Duolink dots with mitochondria. Bar = 10 µm. **B** Quantification of the Duolink-PLA signal in the transfected cells. The number of Duolink dots per individual transfected cells was determined. The values obtained were normalized to cell size. The results are expressed as the ratio between the average normalized number per cell in Bax or Bcl-x_L_ transfected cells to their corresponding control (Flag-pcDNA3) and are presented as mean ± SEM (*n* = 4). (*p < 0.003, paired two-sample Student’s *t* test relative to corresponding control was performed after Log transformation for each treatment) (Dashed line: ratio = 1).

Next, we examined the ability of the Nes2 fragments to interact with anti- and pro-apoptotic Bcl-2 proteins using the Duolink-PLA assay. *Casp9*^*−/−*^ MEFs were transfected with plasmid encoding the His-tagged Nes2 fragments together with plasmid encoding FLAG-Bax, FLAG-Bcl-x_L_ or FLAG-pCDNA3 (control) and GFP-MAO (to identify the transfected cells and the location of the interaction relative to mitochondria). The assay was performed using anti-His and anti-FLAG Abs (Fig. 7A). The Duolink-PLA signal in *Casp9*^*−/−*^ MEFs co-transfected with plasmids encoding Bax or Bcl-x_L_ together with plasmids encoding either His-Nes2 N-terminal or His-Nes2 C-terminal fragments was significantly higher (1.5- 2.3-fold increase) than in cells co-transfected with the control vector together with the Nes2 fragments (Fig. 7B). This suggested that both BH3-like motifs containing Nes2 fragments can interact with Bax and Bcl-x_L_. These interactions are caspase-independent (since they occurred in *Casp9*^*−/−*^ MEFs) and tend to occur in close proximity to mitochondria (since many of the Duolink dots were detected near the GFP-MAO signal) (Fig. 7A). Collectively these results suggest that the Nes2 BH3-like motifs can bind to both pro- and anti- apoptotic proteins and imply that they may act in a similar way as the BH3 domains of the BH3-only proteins.

### BH3-like motif-containing Nes2 fragments can induce apoptosis

**Fig. 8:**
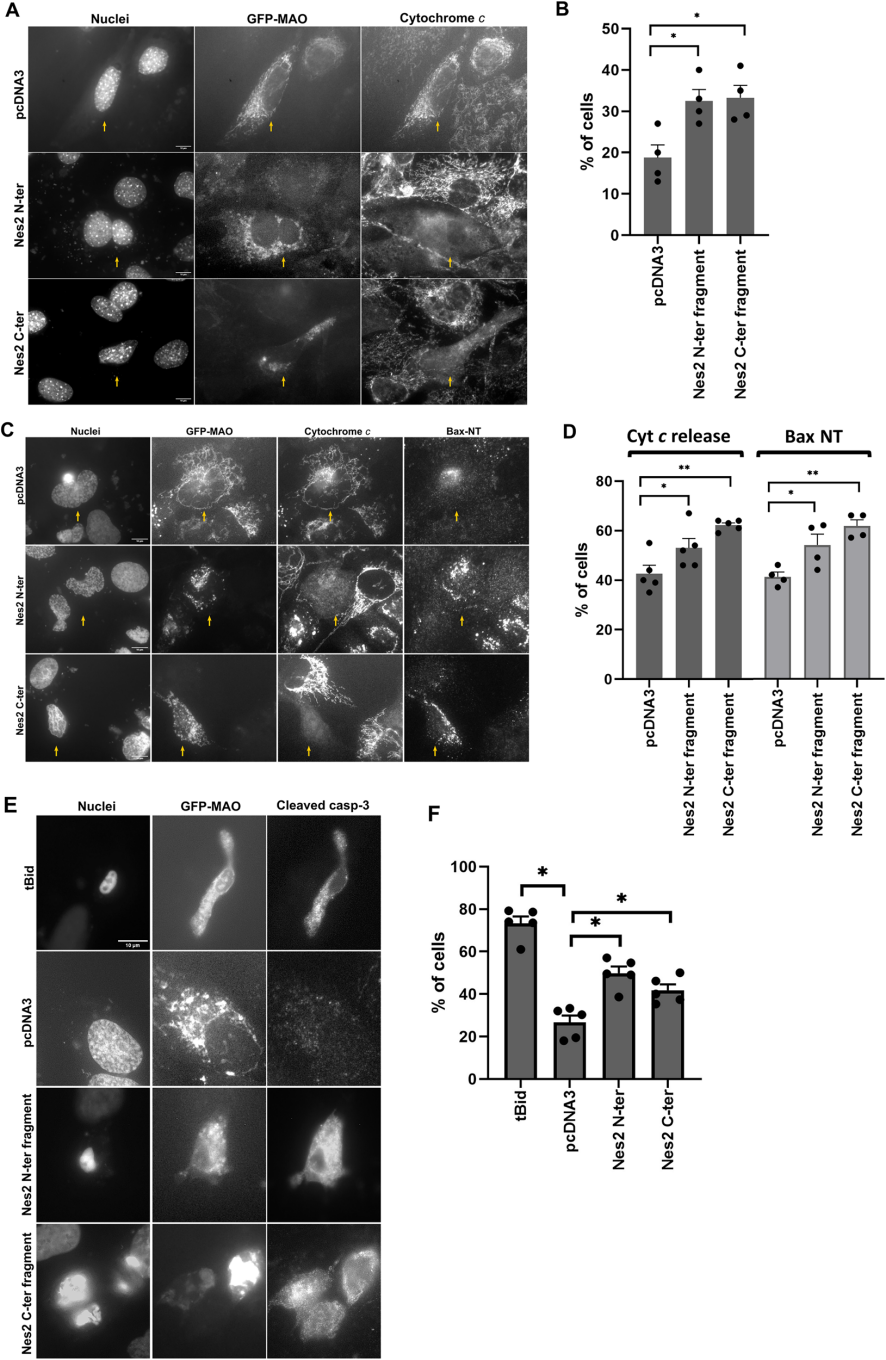
Nes2 fragments promote apoptotic effects. **A**, **B** Cytochrome *c* release in MEFs expressing Nes2 fragments. **A** Representative micrographs of *Casp9*^−/−^ MEFs co-transfected with pcDNA3, His-Nes2 N-terminal (Nes2 N-ter) or His-Nes2 C-terminal (Nes2 C-ter) expression vectors together with GFP-MAO. The micrographs show transfected MEFs (indicated by GFP-MAO signal) with the same field visualized separately for Hoechst dye (Nuclei), GFP-MAO and anti-cytochrome *c* labeling. Bar = 10 µm. **B** Quantification of the percentage of transfected cells exhibiting cytochrome *c* release (a diffused vs. a confined punctate cytochrome *c* distribution pattern) from total transfected cells (at least 100 cells) in each treatment. Dots represent individual experiments. Values are presented as mean ± SEM (error bars) (*n* = 4). (*p < 0.02, two-tailed Student’s *t* test). **C**, **D** Cytochrome *c* release and Bax-NT exposure in U2OS cells expressing Nes2 fragments. **C** Representative micrographs of U2OS cells co-transfected with pcDNA3, His-Nes2 N-terminal (Nes2 N-ter) or His-Nes2 C-terminal (Nes2 C-ter) expression vectors together with GFP-MAO and treated with 20 µM Q-VD-OPH 4 h later. The micrographs show transfected U2OS cells (indicated by GFP-MAO signal) with the same field visualized separately for Hoechst dye (Nuclei), GFP-MAO, anti-cytochrome *c* or anti-Bax NT labeling. Bar = 10 µm. **D** Quantification of the percentage of transfected cells exhibiting cytochrome (Cyt) *c* release (a diffused vs. a confined punctate cytochrome *c* distribution pattern) or Bax-NT signal from total transfected cells (at least 100 cells) in each treatment. Dots represent individual experiments. Values are presented as mean ± SEM (error bars) (*n* = 5 and 4 for cytochrome *c* and Bax-NT, respectively). (cytochrome *c*: *p < 0.05, one-tailed Student’s *t* test **p < 0.0006, two-tailed Student’s *t* test; Bax-NT: *p < 0.05, **p < 0.0007, two-tailed Student’s *t* test). **E**, **F** Cleaved caspase-3 in U2OS cells expressing Nes2 fragments. **E** Representative micrographs of U2OS cells co-transfected with pcDNA3, His-Nes2 N-terminal (Nes2 N-ter) or His-Nes2 C-terminal (Nes2 C-ter) or ^V^tBid expression vectors together with an expression vector encoding GFP-MAO. The micrographs show transfected U2OS cells (indicated by GFP-MAO signal) with the same field visualized separately for Hoechst dye (Nuclei), GFP-MAO and anti-cleaved caspase-3 labeling. Bar = 10 µm. **F** Quantification of the percentage of transfected cells exhibiting cleaved caspase-3 labeling from total transfected cells (at least 100 cells) in each treatment. Dots represent individual experiments. Values are presented as mean ± SEM (error bars) (*n* = 5). (*p < 0.001, two-tailed Student’s *t* test).

Having demonstrated that the BH3-like motif-containing Nes2 fragments can bind Bcl-2 family proteins, we next examined if their expression in cells can promote apoptosis assessed by cytochrome *c* release. To facilitate detection of cytochrome *c* release we employed *Casp9*^*−/−*^ MEFs. These MEFs were transfected with pcDNA3, His-Nes2 N-terminal or His-Nes2 C-terminal expression vectors together with GFP-MAO expression vector and, 24 h later, the percentage of transfected cells (GFP-MAO expressing cells) exhibiting cytochrome *c* release (diffuse vs. defined punctuated patterns) was determined by immunofluorescence (IF) microscopy (Fig. 8A). The percentage of transfected cells exhibiting cytochrome *c* release was significantly higher in the transfected cells expressing the Nes2 N-terminal or Nes2 C-terminal fragments than in cells transfected with control vector (1.8-fold increase for both fragments) (Fig. 8B). These results indicate that the expression of BH3-like motif-containing Nes2 fragments can promote apoptosis in MEFs.

We also examined if the Nes2 fragments can promote apoptosis in human U2OS cells. First, we verified that the fragments can be expressed in transfected U2OS cells (Supplementary Fig. S2). As expected, transfection of U2OS cells with the canonical BH3-only protein tBid promoted cytochrome *c* release and Bax-NT exposure (indicative to Bax activation) demonstrated by IF microscopy (Supplementary Fig. S3A, B, respectively). We then assessed cytochrome *c* release and Bax-NT exposure in transfected cells expressing the Nes2 fragments (Fig. 8C). We then co-transfected U2OS cells with an expression vector encoding GFP-MAO together with expression vectors encoding the Nes2 fragment or pcDNA3 in the presence of Q-VD-OPH (to facilitate detection of cytochrome *c* and Bax-NT signals). The percentage of transfected cells (GFP-MAO expressing) exhibiting cytochrome *c* release or Bax-NT exposure was modestly but significantly higher in cells expressing the Nes2 N-terminal or Nes2 C-terminal fragments compared to cells transfected with a control vector (1.3- and 1.5-fold increase, respectively, for both cytochrome *c* and Bax-NT) (Fig. 8D).

To further assess whether the Nes2 fragments-induced apoptotic pathway can proceed to caspase activation, we examined the appearance of the active form of caspase-3 (cleaved caspase-3) in the transfected cells using anti-cleaved caspase-3 Ab by IF microscopy. U2OS cells were co-transfected with GFP-MAO together with expression vectors encoding the Nes2 fragments or pcDNA3 or with tBid as positive control (in the absence of Q-VD-OPH) (Fig. 8E). The percentage of transfected cells (GFP-MAO-expressing) exhibiting cleaved caspase-3 signal was significantly higher in cells expressing tBid as well as in cells expressing Nes2 N-terminal or Nes2 C-terminal fragments compared to cells transfected with a control vector (2.8-, 1.88- and 1.57- fold increase, respectively) (Fig. 8F). These results show that, like in MEFs, expression of N-terminal or the C-terminal BH3-like motif-containing Nes2 fragments promotes apoptosis in human cells and that the pro-apoptotic effect of Nes2 may be mediated by these BH3-like motifs.

## Discussion

Nes2G is a component of the LINC complex that connects the cytoskeleton and the nucleus. In previous studies we showed that Nes2 can also function as a pro-apoptotic protein via Bcl-2 family proteins [38]. Here we show that endogenous Nes2G can bind both anti- and pro-apoptotic multidomain Bcl-2 family proteins and that this binding occurs via two newly identified BH3-like motifs in Nes2G, located at its N- and C-termini regions that bind to the multidomain proteins of the Bcl-2 family. Furthermore, our results suggest that these motifs mediate the pro-apoptotic function of Nes2.

The Nes2G BH3-like motifs were identified by amino acids sequence alignment to BH3 domains of the pro-apoptotic Bcl-2 family proteins. Both motifs are predicted to adopt amphipathic α helix structure, by themselves and in the context of their immediate surrounding SRs. This is in line with previous findings showing that helicity of BH3 peptides positively correlates with their ability to bind Bcl-2 family proteins [50]. In addition, molecular models predict that both motifs dock onto the canonical groove of Bax and of the anti-apoptotic proteins Bcl-x_L_ and Mcl-1, in a similar manner as Bid and Bim BH3 peptides as well as to Bax’s trigger site. Furthermore, we showed that the C-terminal motif binds to Bax in live cells in the context of the tBid amino acids that surround the BH3 region and that the Nes2 BH3-like motif-containing fragments, SR2-3 and SR50-51 expressed in cells, can bind to pro- and anti- apoptotic Bcl-2 family proteins and promote cytochrome *c* release (indicative of apoptosis).

### Characterization of the Nes2 BH3-like motifs features and their docking potential to multidomain Bcl-2 family proteins

The Nes2 BH3-like N-terminal motif contains all 4 conserved h1-h4 positions of BH3 domains. However, its h3 + 2 position contains asparagine instead of aspartic acid. Notably, previous computational analysis suggested that the presence of asparagine or glutamine instead of aspartic acid at the h3 + 2 position is tolerable in terms of binding to the hydrophobic groove [8, 51]. In line with this assumption, the BH3-like domain of the autophagy protein AMBRA1, which binds Bcl-2, contains glutamine instead of aspartic acid [52]. The C-terminal motif in Nes2 contains the conserved positions h2-h4, but not h1, as well as the conserved aspartic acid residue at position h3 + 2. Despite these deviations from the canonical BH3-domain features, molecular docking simulations predict that both Nes2 BH3-like motifs dock onto the canonical hydrophobic grooves of the effector protein Bax, as well as the anti-apoptotic proteins, Bcl-x_L_ and Mcl-1. Moreover, both motifs are also predicted to dock onto the trigger site of globular full-length Bax as well as to a rear site on Bax’s swapped dimer structure, at the position of α6 in the globular structure. These docking results suggest that the Nes2 BH3-like motifs may act in a similar manner as the BH3 domains of the inducer BH3-only proteins Bim and Bid. It should be noted that given that the Nes2 BH3-like motifs are predicted to localize inside the SRs with their hydrophobic side chains facing inward and hydrophilic side chains facing outward, it is probable that a conformational change in the SRs is needed for the motifs to dock onto the Bcl-2 proteins hydrophobic pockets.

Regarding the prediction that the two Nes2 BH3-like motifs also dock to the trigger site of Bax, we noted that this docking solution is not predicted in the globular structure after removing α9. This observation suggests that the removal of α9 renders the canonical groove more compatible for the binding of the two motifs. Hence, the motifs bind only to this groove. Overall, the docking results predict that the identified BH3-like motifs bind both anti- and pro- apoptotic multidomain Bcl-2 proteins at similar positions as canonical BH3 domains do.

### Binding of the BH3-like motifs to multidomain Bcl-2 family proteins

The predicted binding of the Nes2 BH3-like motifs to BaxΔCTS was confirmed for the C-terminal motif in live cells using qF3 FLIM-FRET and tBid chimeras. The results of these experiments show that when the Nes2 C-terminal motif replaced the Bid BH3 domain, the resulting protein bound to ^C^BaxΔCTS with an apparent *K*_*d*_ comparable to that obtained for ^V^tBid binding to ^C^BaxΔCTS. In contrast, binding to ^C^BaxΔCTS was not detected for a similar Bid chimera containing the Nes2 N-terminal motif. Instead, collisions similar to the chimeras containing scrambled versions of the Nes2 sequences used as controls were observed. As expected for collisions, the FLIM FRET signals fit a linear equation. While collisions were detected, it is difficult to conclude that there is no binding of the Nes2 N-terminal sequence because false negatives are possible due to the stringent distance and dipole alignment requirements of FRET [53, 54].

The binding potential of the Nes2 BH3 motifs to multidomain Bcl-2 family proteins is further supported in the context of their immediate surrounding regions within Nes2G. Accordingly, both Nes2 BH3-like motifs containing fragments expressed in cells were found to interact with exogenously expressed Bax or Bcl-x_L_. The reason that Nes2 N-terminal fragment could bind Bax whereas tBid chimera containing the N-terminal motif did not could be because, in the context of the Nes2 N-terminal fragment, the lack of aspartic acid-arginine salt bridge may be compensated by the amino acids surrounding the Nes2 N-terminal motif.

The finding that the Nes2 N-terminal and C-terminal BH3-like motifs can interact with anti- and pro-apoptotic Bcl-2 family proteins is in line with our previous [39] and current results showing that endogenous Nes2G can interact with Bax [39], Bak, Bcl-2 or Bcl-x_L_ and further implies that the binding is mediated by the Nes2G BH3-like motifs. The molecular docking results predicted binding of both BH3-like motifs to Mcl-1; however, we could not detect binding of endogenous Nes2G to Mcl-1 using the Duolink-PLA approach. This could be due the short half-life of Mcl-1 [55] leading to insufficient amount of Mcl-1 available for the binding. Notably, some of the Duolink-PLA signals corresponding to the binding of endogenous Nes2G and of Nes2 BH3-like motif-containing fragments to Bcl-2 family proteins were found near the mitochondria, the region where the Bcl-2 family protein interaction network occurs. Furthermore, we showed recently that during apoptosis Nes2G translocate to the mitochondria proximity and that this event is associated with mitochondrial dysfunctions such as MOMP [56]. These results further support the notion that interactions between Nes2 and Bcl-2 family proteins via the Nes2 BH3-like motifs occur in cells.

### Nes2 BH3 like motifs and Nes2 isoforms

Nes2G is the only Nes2 isoform that contains both BH3-like motifs. The mechanism whereby the two motifs participate in its binding to Bcl-2 family proteins is currently unknown. The BH3-only protein Bim binds Bax by two regions, the canonical BH3 domain and a BH3-like motif within the C-terminal region [57]. Both these Bim regions can promote Bax activation and only the canonical region can bind anti-apoptotic Bcl-2 proteins. Thus, it is possible that the two Nes2G BH3-like motifs act in a similar manner, i.e., both act simultaneously to induce direct activation of Bax and only one is required to bind the anti-apoptotic proteins. Alternatively, Nes2G may act as a scaffold protein that generates a platform complex that facilitates interactions between Bcl-2 family proteins. In this regard, Bcl-x_L_ forms higher-order complexes that act as a scaffold that binds different BH3 proteins simultaneously, which in turn facilitates Bax activation [58].

Shorter Nes2 isoforms contain only one of the motifs e.g., isoforms ε1, ε2 and γ contain only the C-terminal motif and the p220CH isoform only the N-terminal motif [34]. Since we currently do not know if the two motifs play the same or different roles and how the presence of both motifs in Nes2G affects their function, we cannot predict the function and contribution of each isoform to apoptosis.

### Nes2 BH3-like motif containing fragments can promote apoptosis

Given that Nes2 contains BH3-like motifs that can bind to Bcl-2 family proteins, we hypothesized that these motifs function as BH3 domain of BH3-only proteins that can promote apoptosis. This notion is supported by our results showing that expression of Nes2 BH3-like motif containing fragments in cells can promote cytochrome *c* release in both mouse and human cells. Furthermore, the finding that expression of these fragments also induces Bax-NT exposure as well as caspase-3 activation in U2OS cells further indicates that the apoptotic effect of the Nes2 BH3-like motif containing fragments is mediated as expected by activating Bax. However, the relative contribution of Nes2 as a potential BH3-like protein to Bax activation and subsequent apoptosis most likely depends on the presence of other BH3-only proteins in the cells, its binding affinity to different Bcl-2 family proteins and the nature of the apoptotic stimulus. Additional studies are needed to unravel the biological contribution of Nes2 and its BH3-like motifs in apoptosis.

Overall, our results show that the pro-apoptotic activity of Nes2 is mediated via its BH3-like motifs. Nes2 may therefore act as a pro-apoptotic BH3-only protein, regulating the multidomain Bcl-2 family proteins. These new findings regarding Nes2 further support the notion that the NE acts as a mediator of apoptosis.

## Materials and methods

### Materials

All reagents were purchased from Sigma-Aldrich unless otherwise specified.

### Cell culture

Immortalized wild type (WT) and *Casp9*^*−/−*^ 3T9 mouse embryonic fibroblasts (MEFs) with deletion of the gene encoding caspase-9 [59] were provided by Prof. Christoph Borner (University of Freiburg, Germany). Human osteosarcoma epithelial cell line U2OS was provided by Prof. Ana J. Garcia-Saez (University of Cologne, Germany). 293T HEK cells were provided by Prof. Eran Bacharach (Tel Aviv University, Israel). HCT116 DKO cells expressing mCerulean3-Bax (^C^Bax) or mCerulean3-BaxΔCTS (^C^BaxΔCTS) fusion proteins were generated by lentiviral transduction of HCT116 DKO cells [60], followed by fluorescence-activated cell sorting to isolate pooled colonies of cells stably expressing ^C^Bax or ^C^BaxΔCTS. Expression of ^C^Bax and ^C^BaxΔCTS was further confirmed by immunoblotting using anti-GFP and anti-Bax antibodies. WT MRC5 cells (PMID: 9704922) were used as control. Cells were grown in high-glucose Dulbecco’s modified Eagle’s medium supplemented with 10% heat-inactivated fetal calf serum.

### Plasmids

Expression vectors used in this study were pcDNA3 (Invitrogen), pcDNA3-Flag (Invitrogen), Flag-hBcl-2 [61], Flag-Bcl-x_L_ [61], pEF-Flag-Mcl-1 [62], Flag-Bax [63], GB-mini-nesprin 2G [39], pcDNA3 HA–Bak (HA–Bak, a gift from Gabriel Nuñez, University of Michigan Medical School, Ann Arbor, MI), pCQXIP eGFP-TA MAO [64], PCi (His-Bid) [65], Venus-tBid-pEGFP-C1 (^V^tBid) [66], Venus-tBid-4E-pEGFP-C1 (^V^tBid [BH3-4E mutation]) [49]. His-tagged Nes2 N-terminal and His-tagged Nes2 C-terminal expression vectors were generated by cloning the corresponding cDNAs into pcDNA3 as follows: Total RNA from WT MEFs was prepared with Trizol according to the manufacturer’s protocol. The cDNA encoding Nes2 N-terminal and Nes2 C-terminal fragments was obtained by two-steps RT-PCR. First-strand was synthesized using Revertaid Reverse Transcriptase (Thermo Fisher Scientific) using 5’-CTGCAAGAGGGTCTCAACGG-3’ or 5’- GAGCTCCAGCTGTGTCAGTC-3’ primers for Nes2 N-terminal or Nes2 C-terminal, respectively. The resulting cDNAs were used as templates to amplify internal regions by Q5 High-Fidelity DNA polymerase (Q5® High-Fidelity 2X Master Mix, New England Biolabs) using the following primer sets: forward 5’-GTCACTCGAGATGGCGTGGAGAGCCCAACTGG-3’ and reverse: 5’-GTCAGAATTCCTAATGGATGTATTGTCTGCAC-3’ for Nes2 N-terminal; and forward 5’-GTCACTCGAGATGCGCAAGCAGGAGATTGAAG-3’ and reverse 5’- GTCAGAATTCCTACCAGGTCTCCTCGATTTTC-3’ for Nes2 C-terminal. The PCR products were cloned into the XhoI and EcoRI sites of GB-mini-nesprin2G plasmid. The plasmids obtained were used as template to amplify by PCR the Nes2 cDNAs fused to His tag at their N’ termini using the following primer sets: forward 5’-GTCAGAATTCATGGCTCACCACCACCACCACCACGCGTGGAGAGCCCAACTGG-3’ and reverse: 5’-GTCACTCGAGCTAATGGATGTATTGTCTGCAC-3’ for Nes2 N-terminal, and forward 5’-GTCAGAATTCATGGCTCACCACCACCACCACCACCGCAAGCAGGAGATTGAAG-3’ and reverse 5’-GTCACTCGAGCTACCAGGTCTCCTCGATTTTC-3’ for Nes2 C-terminal. The PCR products were cloned into the XhoI and EcoRI sites of pcDNA3.

### Antibodies

Antibodies (Abs) used for immunofluorescence staining were rabbit anti-His-tag (# ab213204, Abcam) (1:100), mouse anti-cytochrome *c* (# 556432, BD Biosciences) (1:200), mouse anti-Flag M2 Ab (# F1804, Sigma-Aldrich) (1:100), mouse anti-HA (# sc-7392, Santa Cruz) (1:20), rabbit Ab anti-cleaved Caspase-3 (Asp175) (#9664, Cell Signaling Technology) (1:200) and rabbit anti-Bax NT (# ABC11, Merck Millipore) (1:100). For immunoblotting, we used mouse anti-His-tag (# MCA 1396, Bio-Rad) (1:1000), mouse monoclonal anti-GFP (4B10, Cell Signaling Technology) (1:1000), mouse monoclonal anti-human Bax (2D2, Cell Signaling Technology) (1:1000), and rabbit monoclonal anti-GAPDH (14C10, Cell Signaling Technology) (1:1000). For Duolink-PLA, we used mouse anti-Flag M2 Ab (as above), mouse anti-HA (# sc-7392, Santa Cruz) (1:20), rabbit anti nesprin-2G [40] (1:200), and rabbit anti His-tag (# ab213204, Abcam) (1:200).

### Duolink proximity ligation assay (Duolink-PLA)

Duolink-PLA was performed using Duolink in situ PLUS and MINUS probes and Duolink in situ detection reagents FarRed according to manufacturer’s instructions (Sigma-Aldrich). At the end of the procedure, the slides were mounted with a coverslip using Duolink® In Situ Mounting Medium which contains 4,6-diamidino-2-phenylindole, dihydrochloride (DAPI) for nuclear staining. Imaging was performed by fluorescence or confocal microscopy. Duolink signal appears as dots.

### FLIM-FRET

FLIM-FRET on the INO-FHS platform to detect protein-protein interactions in live cells was carried out as previously described [49]. Briefly, 8000 HCT116 DKO (*Bax*^−/−^*;Bak*^−/−^) cells expressing exogenous ^C^BaxΔCTS were seeded into wells of a CellCarrier-384 Ultra Microplate (PerkinElmer). Four hours later, cells were transfected with plasmids to transiently express the specified Venus-tBid or Venus-tBid mutant construct for FLIM-FRET. Four hours after plasmid transfection using the Mirus TransIT-x2 reagent, media in each well was replaced with media containing 10 µM quinoline-VaL-Asp(OMe)-CH2-OPH (Q-VD-OPH, Apex Biotechnology) to remove the Mirus reagent and inhibit caspase activation. Image acquisition was performed on the INO-FHS microscope as described in [49]. Additionally, protein standards of purified mVenus and mCerulean3, as well as fluorescein (10 nM in 0.1 M NaOH) and quenched fluorescein (30 µM fluorescein in 8.3 M NaI and 100 mM Na_2_HPO_4_ at pH10), were added to separate wells in the same 384-well plate for calibration. Image analysis was done using our specialized software [67]. Briefly, regions of interest (ROI) were automatically selected with a watershed algorithm based on the mCerulean3 channel. Pixels from within each ROI were binned before lifetime analysis using the phasor plot approach. Decreases in the lifetime of the donor mCerulean3 due to FRET were quantified and reported as changes in the angular frequency or Δω from Tau1 on the universal circle of the phasor plot, where Tau1 is the fluorescence lifetime of mCerulean3 in cells not expressing Venus (expressing donor protein only). To generate the data for binding curves, the average intensity of the donor (mCerulean3) and the acceptor (Venus) in each ROI was converted to micromolar concentration using the linear equation fitted to the intensity and concentration values of the purified recombinant protein standards for mCerulean3 and Venus respectively. To measure the intensity of the donor independent of FRET, only photons received during the first 350 ps were used [67]. ROIs that were quantified contained a narrow physiological concentration ranging from 1 to 3 µM for mCerulean3 and a wide range of acceptor concentrations (from 0 to 50 µM) for Venus. The data were binned according to the acceptor concentrations to generate binding curves for each technical replicate. The number of ROIs in each bin ranged from at least 20 at the high concentrations of acceptor (high-intensity ROIs are rare) to many thousands at the low acceptor concentrations.

Data points from 3 binding curves from 3 independent replicates were used to generate an average binding curve. This average binding curve is fitted to the Hill equation with a Hill constant (h) of 1 if the maximum Δω (Bmax) determined from the Hill fitting is above the set threshold of 0.05. This requirement ensures there is sufficient dynamic range in the assay. For fitting to a Hill equation, we require that the shape-ratio (s-ratio) of the binding data was equal to or larger than 2 [49], indicating the binding data would fit better to a Hill equation and does not fit well to linear regression. The latter is indicative of collisions rather than binding. Briefly, the s-ratio is calculated as the area under the binding data divided by the area above, where the upper and lower boundaries of the areas are the maximum and minimum Δω values of the data, respectively. Because collisions increase linearly with concentration, the s-ratio is closer to 1 if the binding data fits well to a straight line.

### Transfection

Transfection was performed with TransIT-X2 (Mirus Bio) according to the manufacturer’s instructions. One day before transfection, 10^5^ or 2 × 10^5^ *Casp9*^*−/−*^ MEFs or 2 × 10^5^ U2OS cells were seeded on 18-mm cover slips coated with collagen in 12 well plates (for the experiments aimed to assess protein expression by IF staining). In other experiments, 10^4^ MEFs were seeded on 16-well chamber slides coated with collagen (for the Duolink-PLA experiments) or 3 × 10^6^ 293T HEK cells were seeded on 10 cm plates (for immunoblotting). When indicated, 20 µM Q-VD-OPH was added 4 h after transfection. For the FLIM-FRET experiments, the transfection protocol was described briefly above and full details can be found in Osterlund et al. [68].

### IF microscopy

Transfected *Casp9*^*−/−*^ MEFs or U2OS cells were fixed 24 h after transfection and stained with different Abs and Hoechst 33258 dye or treated with the Duolink-PLA reagents, as described previously [39, 69]. Cells were imaged using a fluorescence microscope (EVOS Cell Imaging Systems, Thermo Fisher Scientific) or confocal microscope (LEICA TCS SP8) using X63 objective lens.

### Immunoblotting

Total cell lysate was prepared from 293T HEK transfected cells [70], HCT116 DKO cells expressing ^C^BaxΔCTS or ^C^Bax, WT MRC5 cells and from His-Bid protein expressing bacteria. Three hundred µg of protein from transfected 293T HEK cells or 0.1 µg of protein prepared from His-Bid expressing bacteria were subjected to SDS-PAGE (12.5%) and electroblotted (1 h, 100 V) onto nitrocellulose membranes in the presence of blotting buffer (186 mM glycine, 25 mM Tris base, 0.08% SDS, 20% methanol). The blots were blocked for 1 h in 10 mM Tris base and 150 mM NaCl containing 5% fat-free milk, then incubated for 24 h at 4 °C with an anti-His-tag primary Ab. Goat anti-mouse (1:10,000) IgG peroxidase conjugate (Jackson Immunoresearch Laboratories) was used as the secondary antibody. The blots were developed using the Immobilon Crescendo Western HRP Substrate (Merck Millipore). For the immunoblots in Supplementary Fig. S1, 10 µg of protein from HCT116 DKO transfected cells or MRC5 cells were subjected to SDS-PAGE. The Abs were diluted 1:1000 in Tris-based saline buffer (TBST) and incubated with the blots overnight at 4 °C. Blots were washed three times with TBST before 2 h incubation at room temperature with 1:20,000 dilution of the secondary HRP-conjugated anti-mouse or anti-rabbit Ab. The blots were developed using Ez-ECL solution (Biological Industries). Bands were detected using the MicroChemi 4.2 Gel Imager (DNR Bio-Imaging Systems Ltd).

### Generation of His-Bid protein in bacteria

His-Bid [65] was expressed in *E. coli* strain BL21-AI. His-Bid protein expression was induced using 0.2% L-arabinose for 3 h at 30 °C. *E. coli* cells were lysed as described previously [71].

### Generation of ^V^tBid/Nes2-BH3 chimeras encoding vectors

To generate plasmids encoding ^V^tBid(Nes2-BH3) chimeras, we replaced the Bid-BH3 region (h0-h4) plus the codons for 3 amino acids downstream (residues 81–100 of Bid) in the plasmid encoding ^V^tBid with the Nes2 N-terminal (encoding 15 amino acids of the BH3-like motif plus 4 amino acids upstream), Nes2 C-terminal (encoding 11 amino acids of the BH3-like motif plus the 7 amino acids upstream and 1 amino acid downstream), or the corresponding sequences encoding scrambled amino acid sequences using FastCloning as described previously [72]. PCR was performed on ^V^tBid using Q5 High-Fidelity DNA polymerase and the following primers: For tBid(Nes2-BH3 N-terminal): forward 5’-GATGACAAGACGAATCAACAACGTTTTGGGTAAAAACATCCAGCCCACACTGGTGAG-3’ and reverse 5’-TGATTCGTCTTGTCATCTCCTCCAGTTTAACAGGAGGTTCTTCCTGACTTTCAGAATC-3’; For tBid(Nes2-BH3 N-terminal-scramble): forward 5’-GGGCCGCAACGTGACCGTGCCGCCGAACATGAAAGAAATCCAGCCCACACTGGTGAG-3’ and reverse 5’-CGGTCACGTTGCGGCCCAGCAGGCGAATTTTTTCGTTTTCTTCCTGACTTTCAGAATC-3’; For tBid(Nes2-BH3 C-terminal): forward 5’-GCAGCTGAAGCAGATGGGCGACCAGCTGATCAAGGCCATCCAGCCCACACTGGTGAG-3’ and reverse 5’-CCATCTGCTTCAGCTGCAGTTTGTTTTCTGTGAATAATTCTTCCTGACTTTCAGAATC-3’; For tBid(Nes2-BH3 C-termnal-scramble): forward 5’-GCTGGAAGATATTAAATTTAAACTGAAAACCCAGATGATCCAGCCCACACTGGTGAG-3’ and reverse 5’-ATTTAATATCTTCCAGCGCCTGCAGCAGGCCGTTCTGTTCTTCCTGACTTTCAGAATC-3’. The PCR products were digested with DpnI and used for FastCloning. All final constructs were verified by DNA sequencing.

### Bioinformatics and computational modeling

The EMBOSS Needle software [41, 42], operated from the EMBL-EBI web service [10.1093/nar/gkac240], was used for pairwise alignments. The MAFFT software [43, 44], operated from the MPI Bioinformatics Toolkit [10.1016/j.jmb.2017.12.007], was used for multiple sequence alignment. Structure prediction was performed by the AlphaFold2 [45] software, operated from ColabFold [46] v1.5.5 with mmseqs2. The prediction was carried out on positions 299-577 (SR1-SR2-SR3) and on positions 5787- 6122 (SR49-SR50-SR51) of mouse Nes2G (UniProt ID: Q6ZWQ0). Blind protein-peptide docking was performed using PatchMAN [48] software with rigid protein backbone and with the docked peptide specified by sequence. The results of Fig 3. include the top ten solutions. The proteins structures were obtained from the Protein Data Bank (PDB) accession numbers: 5W62, 4BD2, 1PQ1, 2NL9.

### Statistical analysis

Statistical significance was determined using one-tailed or two-tailed Student’s *t* test or paired two-sample Student’s *t* test after Log transformation. Values of *p* < 0.05 were considered statistically significant. Data were expressed as mean values ± SEM.

## Supplementary information

Supplementary Figure legend

Figures S1-3

Figure S4 related to Figure 6

## Data Availability

Original data are available upon request. The full length uncropped original western blots are shown in the ‘Supplementary Material’.
