# Supplementary material for: Nesprin-2 contains BH3-like motifs that can promote cell death
Source: Cell Death Discov. 2025 Jun 3;11:263. doi: 10.1038/s41420-025-02534-5 (PMC12134178; doi:10.1038/s41420-025-02534-5)
Supplement: Supplementary file 1 — Supplementary Figure legend [file 41420_2025_2534_MOESM1_ESM.docx]

**Nesprin-2 contains BH3-like motifs that can promote cell death**

Hila Zohar^1^, Amit Kessel^2^, Liora Lindenboim^1^, Dang Nguyen^3,4^, Nir Ben-Tal^2^, Gregg G. Gundersen^5^, Howard J. Worman^5,6^, David W. Andrews^3,4,7^ and Reuven Stein^1^*

^1^Department of Neurobiology, School of Neurobiology, Biochemistry and Biophysics, George S. Wise Faculty of Life Sciences, Tel Aviv University, Tel Aviv 69978, Israel.

^2^Department of Biochemistry and Molecular Biology, School of Neurobiology, Biochemistry and Biophysics, George S. Wise Faculty of Life Sciences, Tel Aviv, Israel.

^3^Department of Medical Biophysics, Faculty of Medicine, University of Toronto, Toronto, Canada.

^4^Biological Sciences Platform, Sunnybrook Research Institute, Toronto, Canada.

^5^Department of Pathology and Cell Biology, Vagelos College of Physicians and Surgeons, Columbia University, New York, NY 10032, USA.

^6^Department of Medicine, Vagelos College of Physicians and Surgeons, Columbia University, New York, NY 10032, USA.

^7^Department of Biochemistry, Faculty of Medicine, University of Toronto, Toronto, Canada.

*Corresponding author: R. Stein, Department of Neurobiology, School of Neurobiology, Biochemistry and Biophysics, George S. Wise Faculty of Life Sciences, Tel Aviv University, Tel Aviv 69978, Israel

**Supplementary Figures Legend**

**Supplementary Figure S1**. **Expression of ^C^BaxΔCTS in HCT116 DKO verified by immunoblotting.** Cell lysates from three cell lines (MRC5, ^C^Bax HCT116 DKO, and ^C^BaxΔCTS HCT116 DKO) were diluted to the same protein concentration of 2 mg/mL before loading into the indicated lanes of the SDS-PAGE gels for immunoblot. Ab dilutions are shown above the lanes on the immunoblots. Left panel: Anti-GFP antibodies were used to detect the presence of mCerulean3 in the cell lysate. Luminescence signals coming from HRP were present only in lane 2 and lane 3, corresponding to the two HCT116 DKO cell lines expressing mCerulean3-full-length Bax fusion protein (lane 2) and mCerulean3-BaxΔCTS (lane 3). The small shift in the molecular weight of ^C^BaxΔCTS compared to ^C^Bax is due to truncation of the former. This shift is also visible in the second blot (middle panel) where anti-Bax antibodies were used to detect the presence of Bax in the cell lysate. The bands indicated by the box migrate to the same position in A and B, indicating that the proteins contain both mCerulean3 and Bax. The MRC5 cell lysate was blotted to detect endogenous Bax in lane 1 and confirms that the DKO cells do not express Bax. The right panel shows a separate immunoblot probed with anti-GAPDH antibody run in parallel as a loading control for the three cell lines.

**Supplementary Figure S2.** **Expression of Nes2 N-terminal and Nes2 C-terminal fragments in U2OS cells.** Representative IF micrographs (n = 2) of U2OS cells transfected with His-Nes2 N-terminal (Nes2 N-ter) or His-Nes2 C-terminal (Nes2 C-ter) expression vectors together with GFP-MAO (with 20 µM Q-VD-OPH) stained with anti-His Ab and Hoechst dye. The IF micrographs show the same field visualized separately for Hoechst dye (Nuclei), His Ab (Nes2 fragment) and GFP-MAO labeling. Bar = 10 µm.

**Supplementary Fig. S3.** **tBid promotes apoptotic effects in U2OS.** U2OS cells were co-transfected with pcDNA3, or ^V^tBid expression vector together with GFP-MAO, followed by staining with anti-cytochrome *c* and anti-Bax NT Abs. (A-B) Representative micrographs of transfected cells (left panels) and quantification (right panels) of the percentage of transfected cells exhibiting cytochrome (Cyt) *c* release (A) or Bax-NT signal (B) from total transfected cells (at least 100 cells) in each treatment. Dots represent individual experiments. Values are presented as mean ± SEM (error bars) (n = 3 for cytochrome *c* and Bax-NT, respectively). (cytochrome *c*: **p* < 0.0001, two-tailed student’s *t* test; Bax-NT: **p* < 0.005, two-tailed student’s *t* test). The micrographs show transfected U2OS cells (indicated by GFP-MAO signal) with the same field visualized separately for Hoechst dye (Nuclei), GFP-MAO and anti-cytochrome *c* (A) or Bax-NT (B) labeling. Bar = 10 µm.

**Supplementary Fig. S4. Full and uncropped immunoblot corresponding to Fig. 6B.**
