# Supplementary material for: Nesprin-2 contains BH3-like motifs that can promote cell death
Source: Cell Death Discov. 2025 Jun 3;11:263. doi: 10.1038/s41420-025-02534-5 (PMC12134178; doi:10.1038/s41420-025-02534-5)

Supplementary Figure 1

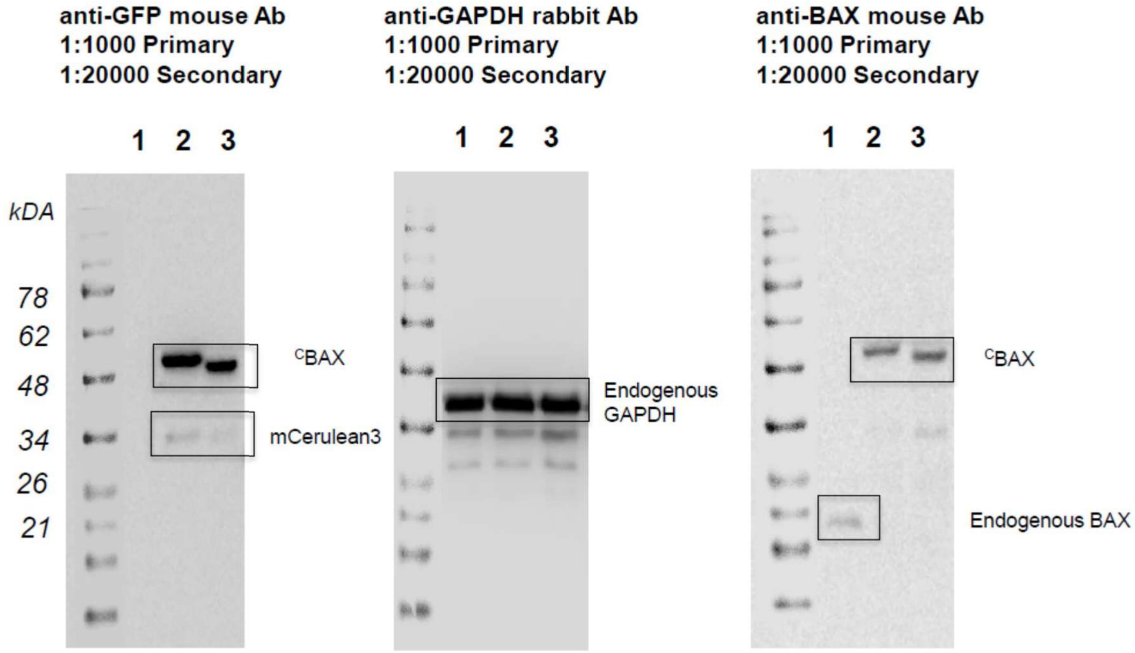

Lane #

1) **MRC5 WT cells**
2) **HCT116 DKO expressing ^{C}Bax**
3) **HCT116 DKO expressing ^{C}Bax∆CTS**

Supplementary Figure 2

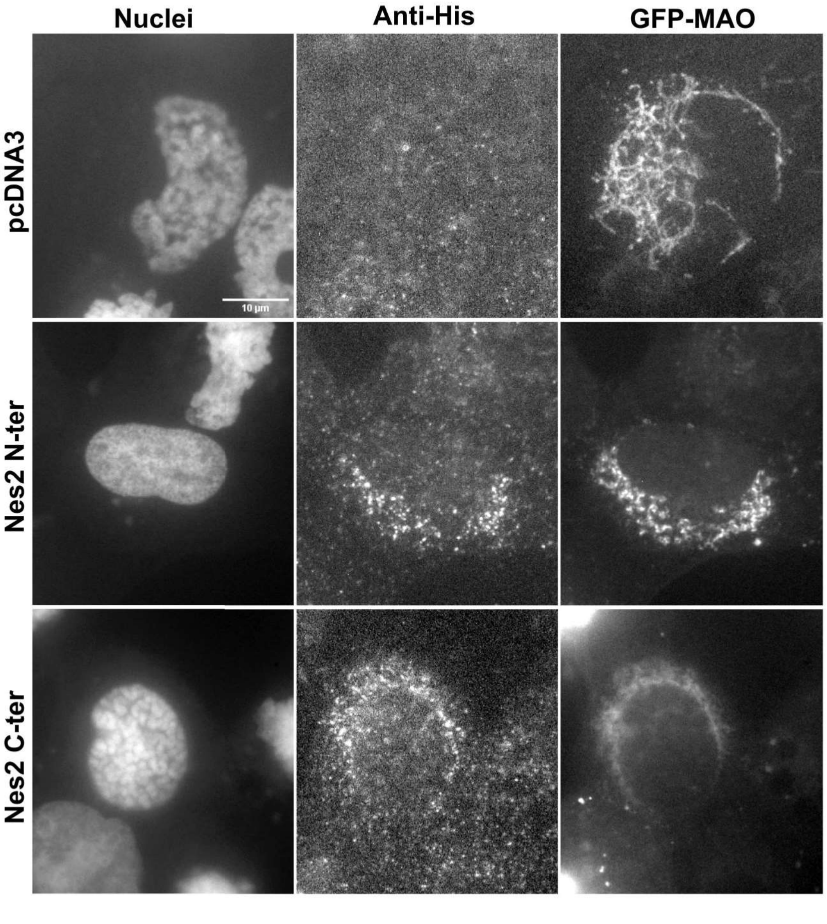

Supplementary Figure S3

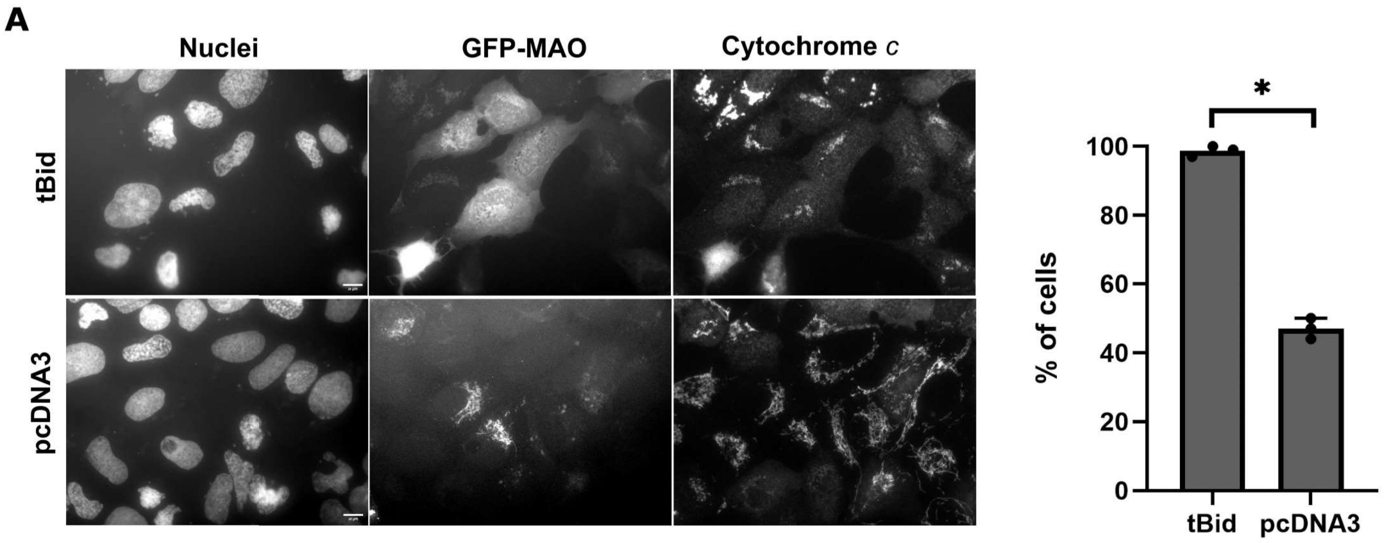

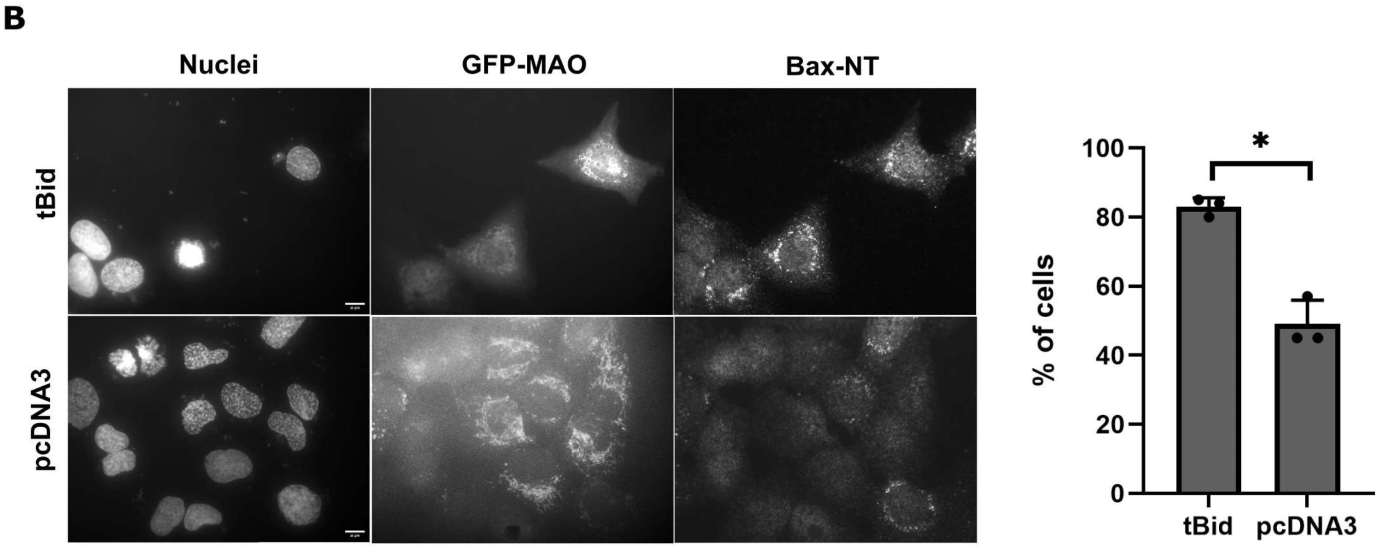

Supplement: Supplementary file 2 — Figures S1-3 [file 41420_2025_2534_MOESM2_ESM.pdf]
