# Supplementary figures and images for: Nesprin-2 contains BH3-like motifs that can promote cell death
Source: Cell Death Discov. 2025 Jun 3;11:263. doi: 10.1038/s41420-025-02534-5 (PMC12134178; doi:10.1038/s41420-025-02534-5)

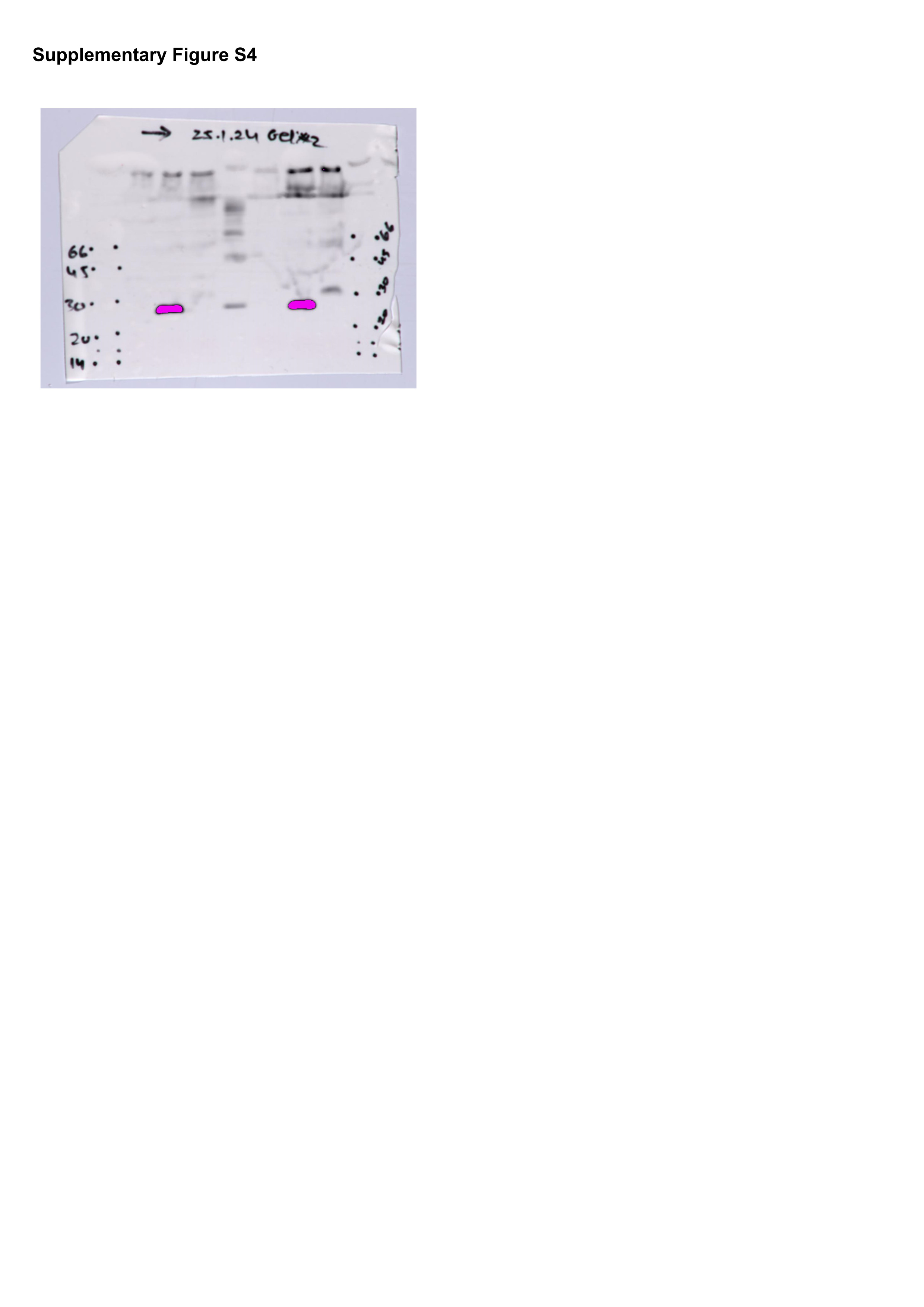

Supplement: Supplementary file 3 — Figure S4 related to Figure 6 [file 41420_2025_2534_MOESM3_ESM.tif]
